# Robustness of cell cycle control and flexible orders of signaling events

**DOI:** 10.1038/srep14627

**Published:** 2015-09-30

**Authors:** Hao Zhu, Yanlan Mao

**Affiliations:** 1 Bioinformatics Section, School of Basic Medical Sciences, Southern Medical University, Shatai Road, Guangzhou, 510515, China; 2 MRC Laboratory for Molecular Cell Biology, University College London, Gower Street, London WC1E 6BT, UK

## Abstract

The highly robust control of cell cycles in eukaryotes enables cells to undergo strictly ordered G1/S/G2/M phases and respond adaptively to regulatory signals; however the nature of the robustness remains obscure. Specifically, it is unclear whether events of signaling should be strictly ordered and whether some events are more robust than others. To quantitatively address the two questions, we have developed a novel cell cycle model upon experimental observations. It contains positive and negative E2F proteins and two Cdk inhibitors, and is parameterized, for the first time, to generate not only oscillating protein concentrations but also periodic signaling events. Events and their orders reconstructed under varied conditions indicate that proteolysis of cyclins and Cdk complexes by APC and Skp2 occurs highly robustly in a strict order, but many other events are either dispensable or can occur in flexible orders. These results suggest that strictly ordered proteolytic events are essential for irreversible cell cycle progression and the robustness of cell cycles copes with flexible orders of signaling events, and unveil a new and important dimension to the robustness of cell cycle control in particular and to biological signaling in general.

In the past decades experimental and computational biologists have tried to unveil the properties and control mechanisms of robust molecular signaling that are believed to be functionally important. The robustness of cell cycle control system in eukaryotic cells, due to its periodic activity and biological importance, has been intensively investigated. Most eukaryotic cells undergo four phases to finish a round of division. When growth factors drive a cell to enter into the G1 phase and pass through the restriction point (a point beyond which mitosis completion is ensured independently of the presence of growth factors), progression of the following S, G2, and M phases is controlled by the sequential activation of a family of cyclin/Cdk complexes (abbreviations shown in Fig. 1B are used hereafter, and italics beginning with a normal letter designate genes). In 1989, Hartwell and Weinert expressed that “The events of the cell cycle of most organisms are ordered into dependent pathways in which the initiation of late events is dependent on the completion of early events”^1^. This viewpoint stresses the importance of the order of signaling events for the robust control of cell cycles. However, so far in all experimental and computational investigations robustness is evaluated by examining if concentrations of targeted molecules in a cell or a mathematical model are sensitive to perturbations or parameters. This method bears two drawbacks. First, signaling happens in diverse contexts, and molecular responses to perturbations and parameters may reveal more about adaptiveness than robustness of the control mechanisms. Second, both protein concentrations and model parameters (such as binding affinity) are difficult to be accurately measured in experiments, leaving the order of, and dependency between, events still unclear. While Hartwell and Weinert defined that “control mechanisms enforcing dependency in the cell cycle are here called checkpoints”, it remains unknown whether the dependency is enforced by merely checkpoints or also by other events. In brief, the nature of the robustness of cell cycle control is still inadequately understood.

To uncover the control mechanisms of the accurate timing and order of cell cycle phases, considerable mathematical models have been built. In early 1990 s, pioneer theoretical studies examined the negative and positive feedbacks in the M phase^2^,^3^,^4^. In late 1990 s, a concise model was developed to simulate the four phases^5^. Since then, further studies have focused on either molecular details such as protein translocation between cytoplasm and nucleus^6^ and multisite phosphorylation^7^, or mechanistic properties such as robustness of cell cycles^8^ and mode lock behavior^9^. While it is well accepted that the control mechanisms in cells share common features^10^, these studies led to different hypotheses, especially the clock and domino hypotheses^11^, to explain strictly ordered G1/S/G2/M phases, and there are points arguing for and against the importance of regulated proteolysis of cyclins and CDKs^12^,^13^,^14^. Moreover, while it is known the four phases are strictly ordered, it remains unclear to what extent signaling events should be ordered. The novel findings revealed by a recent experimental study^15^ suggest that the timing of some events denies the previous understanding (they occur earlier than previously assumed). Thus, to develop new computational methods and models to explore signaling events is important, and a good hypothesis obtained should explain how orders of signaling events ensure both strictly ordered cell cycle phases and cell’s adaptive responses to regulatory signals.

The cell cycle control mechanism contains multiple coupled positive and negative feedbacks, including the well annotated Rb-E2F and Wee-Stg-CDKB feedbacks that control G1/S and G2/M phases^16^. In *Drosophila*, mutations of cyclin D and its sole partner Cdk4 have little effect in any tissue^16^, and the cyclin E/Cdk2, cyclin A/Cdk1, and cyclin B/Cdk1 complexes comprise the most parsimonious system to drive the G1/S/G2/M transition (reviewed in^17^,^18^). In *Drosophila* ectopic expression of *cycE* and *stg*, the rate-limiting positive regulators for G1/S and G2/M progression, truncates G1 and G2, yet the total length of cell cycle is largely maintained^19^. This indicates a regulatory connection between G1/S and G2/M phases. Reis *et al.* examined two explanations for the observed connection, proposed a long-range feedback between G1/S and G2/M, and suggested that the feedback “is an active mechanism that derives from inherent properties of the cell cycle control apparatus”^20^. But, no quantitative examination of the feedback has been made. Cell cycle activities are frequently tuned by developmental signals, and especially, cell divisions need to be stopped at precise times in developing tissues. In the developing *Drosophila* eye, a burst of *stg* expression in a band of cells (the morphogenetic furrow) makes most cells arrest in G1^21^, but a few cells escape the G1 arrest to enter into a new round of division after cells past this round of mitosis^22^. It remains not accurately known how sustained cell cycles are stopped and how G1 arrest is escaped. Since the proposed long-range feedback between G1/S and G2/M enables the cell to adaptively shorten or elongate G1/S and G2/M phases, it should be physiologically important. Also, as seen in *Drosophila* wing, when cell cycles are perturbed by ectopic expression of *dap, wee*, and *myc*, this mechanism enables cells to maintain the normal rate of division^20^. In contrast, cancerous cells often do not make compensatory changes^23^.

Most mathematical models of cell cycle control focused on the robust progression of either the G1/S or G2/M phases and examined robustness only upon oscillating protein concentrations^7^,^24^,^25^. Flexible orders of signaling events, which should be an important aspect of robustness, have never been explored. Based on abundant experimental findings about how dividing cells respond to developmental signals, we have developed a cell cycle model containing the most essential three Cdk modules to simulate G1/S/G2/M phases. The model was built upon key molecular interactions observed in mammalian cells and in *Drosophila* cells. The reasons for building such a model that integrates observations in mammals and *Drosophila* are that available observations in neither species are sufficient to formulate full and detailed feedbacks, the cell cycle control machinery is highly conserved in metazoans, and *Drosophila* development shows rich phenotypes.

For the first time, the model produces not only oscillating protein concentrations but also periodic signaling events. Moreover, compared with previous models (including the recent ones^26^,^27^,^28^), it includes two Cdk inhibitors (Dap and Rux) that target different Cdk complexes and antagonistic E2Fs (E2F1 and E2F2) that function as positive and negative regulators. Simulations focus on the distinct roles of Dap, Rux, E2F1, E2F2, Stg, and Wee in cell cycle control and generate results indicating that the E2F1-centered long-range feedback can regulate G1/S and G2/M phase compensation. More notably, simulations reveal that not all signaling events are essential and equally robust and that events of APC- and Skp2-conducted proteolysis of Cdk complexes should occur in a strict order. We postulate that while irreversibility of cell cycle phase progression is the consequence of systems-level feedbacks^29^, as originally proposed^30^ and recently examined^28^ the highly ordered proteolytic destruction of Cdk complexes is likely to be the most essential events. Our results indicate that flexible lengths of cell cycle phases and flexible orders of signaling events are intrinsically associated and are key features of robust cell cycle control.

## Results

### Parameterize the model upon signaling events

**Figure 1:**
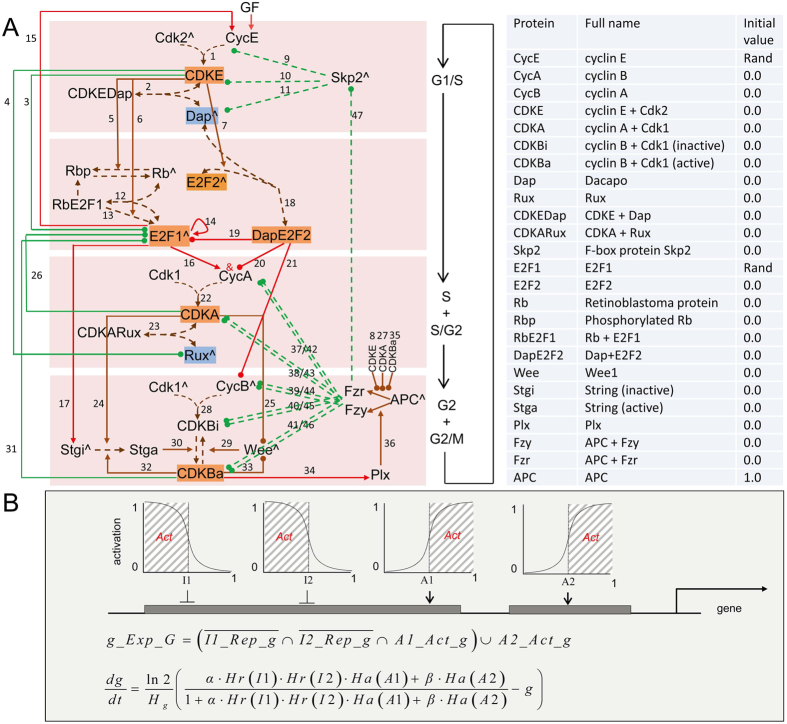
The cell cycle model and definition of events. (**A**) Molecular interactions. The left panel shows protein interactions indicated by numbered links. Specifically, E2F1 in mammals is phosphorylated by CDKE and CDKB^20^ and CDKA^60^,^61^ for degradation, but in *Drosophila* is specifically destructed by CRL4Cdt2 periodically^62^,^47^. How CRL4Cdt2 is down-regulated after S phase is unclear^63^. Recent studies reveal that Cdt2 in CRL4Cdt2 is degraded by SCF^Fbxo11^ and this degradation is prevented by phosphorylation of Cdt2 by CDKs^64^. Thus CDKs’ roles in preventing Cdt2 degradation may promote E2F1 destruction by CRL4Cdt2, agreeing with the initial observation that CDKs mediate E2F1 destruction. Directly or indirectly, we let CDKs mediate E2F1 degradation. Proteins with the symbol ^ indicate that they have a constant synthesis rate. Solid red and brown links ending with an arrow or a dot indicate activation or repression. Dashed brown links ending with bi- or uni-directional arrow(s) indicate binding and unbinding between proteins or protein transformation. Solid and dashed green links ending with a dot indicate degradation via ubiquitination by Skp2/APCFzy/APCFzr. ‘&’ means combined condition. The right panel shows abbreviations, full names, and initial values (Supplementary Table 4) of 25 proteins and complexes described by 25 ordinary differential equations. (**B**) Signaling events are defined upon nonlinear molecular interactions. Shown is expression of gene *g* repressed by two inhibitors I1 and I2 and activated by two activators A1 and A2 independently at two enhancers. Repressive and activating Hill functions (Hr and Ha in the equation of *dg/dt*), with half-maximal activating/inhibiting coefficients, describe how I1, I2, A1, and A2 nonlinearly regulate *g* (functional concentration ranges of these regulators are indicated by shadowed areas). When I1, I2, A1, or A2 exceeds their half-maximal activating/inhibiting coefficients, event *I1_Rep_g, I2_Rep_g, A1_Act_g*, or *A2_Act_g* occurs. When *I1_Rep_g* and *I2_Rep_g* are absent and *A1_Act_g* is present at an enhancer, or *A2_Act_g* is present at the other enhancer, *g_Exp_G* occurs.

To investigate cell cycle control at the systems level, the interactions between 25 most essential proteins and their complexes are integrated into 25 differential equations based on experimental findings (reviewed in^17^,^18^,^31^) (Fig. 1; Supplementary Methods). Specifically, the interactions between E2F1 and CDKE/CDKA/CDKB are based on the findings that E2F1 regulates, and is regulated by, multiple Cdk complexes and that these regulations may form a feedback between G1/S and G2/M progression^20^. Handle of the negative E2F protein E2F2 (E2F4 in mammals), which has been ignored in all previous models, is based on that E2F4 needs p27/p130 (but not Rb) to co-locate to E2F-responsive promoters of target genes, which include *B-myb, CycA, Cdk1*, and *E2F1*^32^,^33^.

**Figure 2:**
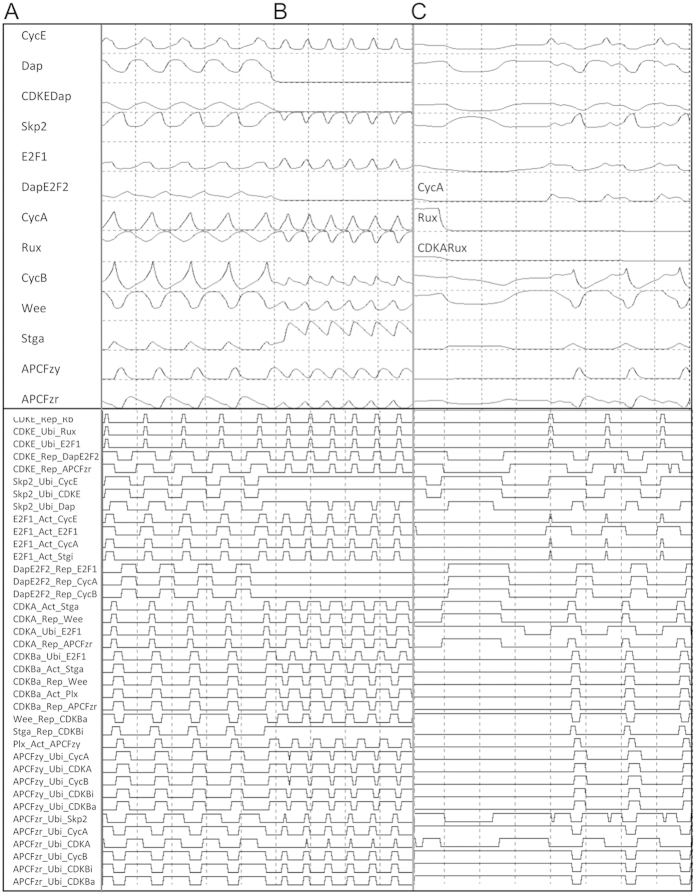
Computed protein concentrations and reconstructed signaling events. d_X_ and s_X_ are protein X’s synthesis and decay rates, and a_XY_ is the parameter in a Hill function describing how X activating Y nonlinearly. Each panel’s top and bottom parts show protein concentrations (between 0.0–1.0) and signaling events (indicated by elevated line segments). (**A**) Under the default parameters all signaling events occur periodically. (**B**) If the decay rate of active Stg is reduced (d_Stga _= 1.0→0.3) cell cycle is arrested at G1 (not shown); but, if the reduction is accompanied by the removal of Dap from the system by setting the synthesis rate of Dap 0.0 (d_Stga _= 1.0→0.3 & s_Dap _= 1.0→0.0) escape of G1 arrest occurs. (**C**) If degradation of E2F1 mediated by CDKA occurs earlier (a_CDKAE2F1 _= 0.14→0.07) cell cycle does not occur, but if this change is accompanied by the removal of Rux (a_CDKAE2F1 _= 0.14→0.07 & s_Rux _= 1.0→0.0) cell cycles are recovered (see the sharp down of Rux in simulation, proteins different from those in (**A**) and (**B**) are marked). (BC) were captured continuously as the parameters changed in simulation (see the sharp down of Dap). In (AB) the wild fluctuations of the first few cycles are caused by the initial conditions and changed parameter, not by noises.

Molecular interactions are defined in the model and captured in simulations, including *A_Act_B* (A activates B), *A_Ubi_B* (A ubiquitinates B), and *A_Rep_B* (A represses B) (Figs 1 and 2). Upon experimental observations the model was parameterized to first produce oscillating protein concentrations and second to produce periodic signaling events (Fig. 2A; Supplementary Table 1). For most parameters, a large range allows them to generate oscillating protein concentrations, indicating robustness of the model (Supplementary Table 2). The ranges of some E2F2-, Skp2-, E2F1-, and CDKE-related parameters are narrow. To make the model also generate periodic signaling events makes parameters biologically more qualified. Notice that while many parameter settings enable the model to produce oscillating protein concentrations, much fewer enable it to produce all periodic signaling events. Since the start of G1, S, G2, and M phases is featured by the maximal value of APCFzy, the rise of CycA, the rise of CDKBa, and the maximal value of CDKBa, we let the start of G1/S/G2/M be marked by the stop of *CDKBa_Ubi_E2F1*, the stop of *DapE2F2_Rep_CycA* and the start of *E2F1_Act_CycA*, the start of *Stga_Rep_CDKBi*, and the start of *APCFzy_Ubi_CDKBi* and *APCFzy_Ubi_CDKBa*, respectively (Fig. 1).

The model can produce cell cycles with varied concentrations of the growth factor GF (a parameter in CycE’s equation) (Fig. 2A) and with the absence of Dap, Rux, or E2F2, respectively (Supplementary Figure 1). Low levels of GF, over a sufficient period, can turn E2F1 on and activate a cell cycle, whereas high levels of GF needs less time to reach the result. If GF is removed before and after a key time point in G1, cells either return back to a stable state or complete a cycle. The feedback between Rb and E2F1 forms a bistable switch enabling graded GF inputs to be converted into all-or-none E2F1 responses. The Cdk1-Wee-Stg system, with positive and double-negative feedbacks, is also bistable. These results are consistent with previous findings^26^,^34^,^35^,^36^,^37^. To include two Cdk inhibitors and antagonistic E2F proteins, which was not seen in previous models, is important for more realistically examining cell cycle control.

### The E2F1-centered feedback between CDKE and CDKB influences the timing, duration, and order of signaling events

**Figure 3:**
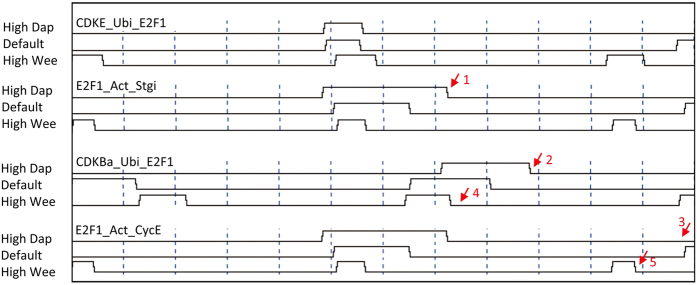
Changed timing, duration, and order of signaling events caused by the E2F1-centered long-range negative feedback. The stop of *CDKBa_Ubi_E2F1* marks the start of G1. “High Dap” and “High Wee” indicate s_Dap _= 1.0→1.05 and s_Wee _= 1.0→1.8. Numbered arrows indicate events of interest. 1: E2F1 functions for a longer time to activate Stgi into Stga. 2: Fewer CDKE causes a delayed G1. 3: E2F1 needs a longer time to trigger *E2F1_Act_CycE* in next round. 4: Fewer CDKBa phosphorylates E2F1 for ubiquitination for a shorter period. 5: More accumulated E2F1 quickly triggers *E2F1_Act_CycE*.

After the above examinations, we then examined cell cycle phase compensation, an important indication of robustness and adaptiveness of cell cycle control. Reis and Edgar observed that when the G1 Cdk inhibitor Dap is overexpressed, G1/S are elongated, G2/M are shortened, and the total cell cycle length is maintained, and hypothesized that E2F1, which is down regulated by CDKE and CDKB, is the key regulator of cell cycle phase compensation^20^. To examine this hypothesis, we simulated *dap* overexpression. When s_Dap _= 1.0→1.05 (“High Dap” in Fig. 3) (s_X_ and d_X_ indicate X’s synthesis and decay rate, respectively), Dap turns more CDKE into the inactive CDKEDap, and fewer CDKE causes a delayed G1 and phosphorylates fewer E2F1 for ubiquitination. Accumulated E2F1 subsequently functions for a longer time to activate Stgi into Stga. Since more Stga quickly activates more CDKBa, the G2/M phases become shortened and CDKBa phosphorylates E2F1 for ubiquitination for a longer period. In the next round, E2F1 needs a longer time to accumulate to the threshold level to activate CycE (Fig. 3).

Reis and Edgar also found that when the Cdk1 inhibitory kinase Wee is overexpressed the G2/M phases are elongated, following shortened G1/S phases^20^. Our model shows that, when s_Wee _= 1.0→1.8 (“High Wee” in Fig. 3), increased Wee deters the transition of CDKBi to CDKBa and causes the elongated G2/M phases. Fewer CDKBa thus phosphorylates E2F1 for ubiquitination for a shorter period. When cells enter into the next G1 phase, the more accumulated E2F1 quickly activates CycE, resulting in a shortened G1/S (Fig. 3). These simulations indicate that when CDKE or CDKB becomes high to facilitate G1/S or G2/M transition, the negative regulation of E2F1 by CDKE and CDKB makes the down-regulated E2F1 deter the subsequent G2/M or G1/S transition (Table 1). If the mediated degradation of E2F1 by CDKE and CDKB is absent as in all previous models^7^,^26^, cell cycle phase compensation does not occur.

**Figure 4:**
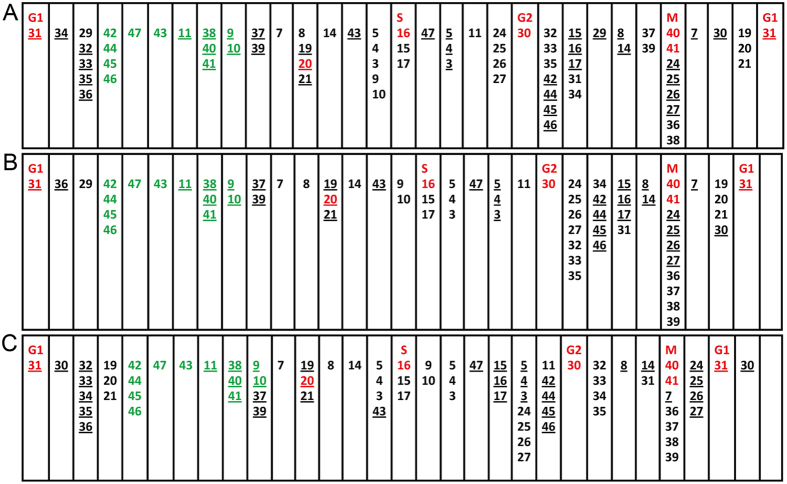
The order of signaling events in a full cell cycle under different conditions. Numbers without and with underlines indicate the start and stop of events (see Fig. 1), respectively. Red numbers indicate G1/S/G2/M events. Compared with the events in the Cdk1-Wee-Stg feedbacks (including 24, 25, 29, 30, 32, and 33), and events in the Rb-E2F1 feedback (including 5, 7, 14, and 15), events of protein ubiquitination (in green, from the column 42/44/45/46 to the column 9/10), have a robust order in the three situations. (**A**) Under default parameters. (**B**) *dap* overexpression (s_Dap _= 1.0→1.05), and (**C**) *wee* overexpression (s_Wee _= 1.0→1.8).

Cell cycle phase compensation influences not only the timing and duration of molecular interactions, but also their orders. Simulations demonstrate that the order of events indicating the onset of G1/S/G2/M remains the same, but the order of other events is changed. For example, events in the Rb-E2F1 feedback and the Cdk1-Wee-Stg feedbacks shift their relative order when *dap* and *wee* are overexpressed (Fig. 4). Thus, not all signaling events are strictly ordered. Inessential events can have flexible orders and essential or robust events have strict orders. This is comparable to the classification of parameters into “essential” ones and “modulatory” ones, which make a cell cycle model behaves differently to perturbations^38^.

### Negative regulators play distinct roles in cell cycle arrest and escape of arrest

We next examined cell cycle arrest and escape of cell cycle arrest, which occur widely in tissue and organ development. To explain the findings that cell divisions in the *Drosophila* eye are arrested in G1 in cells expressing *stg* and that Rux is essential for the cell cycle arrest^21^,^39^, Thomas *et al.* proposed a two-step process. First, cells in G1 are inhibited by some factor from entering into the S phase; second, cells in G2 are driven by Stg to go through M phase, and after reentering into G1 they are prevented from reinitiating a new cell cycle^21^. This explanation suggests that for G1 arrest the repression of CycE and E2F1 is required. The S-phase Cdk inhibitor Rux was later found to physically associate with CDKA to facilitate G1 arrest^40^, but this does not adequately explain how E2F1 is repressed and why the expression of *stg*, a G2/M activator, causes cell cycle arrest.

To quantitatively explore Stg-induced cell cycle arrest, we simulated the impact of high level active Stg (d_Stga _= 1.0→0.3). The increased Stg causes quicker accumulation and higher level of CDKBa, which directly produces three effects: shortened G2/M phases, decreased E2F1 level (due to CDKBa-mediated E2F1 degradation), and increased APCFzy level (due to CDKBa-activated Plx). Subsequently, because of the significantly decreased E2F1, activation of CycE and CycA does not occur, and because of the increased APCFzy, CycA is further decreased. The CDKE-E2F1-CDKB feedback thus enables the high level of Stg, via degradation of E2F1 by CDKB, to make cell cycles arrested (Supplementary Figure 2).

To examine the role of Dap in the arrest of cell proliferation^41^,^42^, we simulated the impact of Dap with s_Dap _= 1.0→0.0 together with d_Stga _= 1.0→0.3. We found that in this situation cells re-entered into G1, and in a rapid pace (Fig. 2B), that is, the absence of the CDKE inhibitor Dap enables cells to escape from G1 arrest. Meanwhile, the absence of the CDKA inhibitor Rux and the absence of the negative E2F protein E2F2 do not have the role. In comparison, high levels of Dap and E2F2 alone can cause cell cycle arrest, but high levels of Rux are more tolerable (Table 2, Supplementary Figure 3–5).

Increased Stg needs Rux to arrest cells in G1, otherwise cells in *rux* mutant will bypass G1 and be arrested in S phase^21^,^40^. In simulations the removal of Rux does not enable the escape of G1 arrest under the condition d_Stga _= 1.0→0.3 (Table 2), but can drive cell cycle progression under some conditions. For example, if a_CDKAE2F1 _= 0.14→0.07 cell cycle fails to occur; but if Rux is removed (a_CDKAE2F1 _= 0.14→0.07 + s_Rux _= 1.0→0.0) cell cycles are recovered (Fig. 2C). In this case, as no CDKA is bound by Rux, swinging CycA gradually produces enough oscillating CDKA for entering into the S phase (Fig. 2C). These results reveal distinct roles of Dap, Rux, and E2F2. These three negative regulators are dispensable under the default parameters and probably under many physiological conditions, but enable the core control system to respond flexibly to regulatory signals. Together, Fig. 2B,C suggest that functions of Dap and Rux are distinct and context-dependent. While they may be dispensable as extra regulators of CDKE and CDKA, Dap influences cell cycles more significantly because it inactivates CDKE and activates E2F2, while Rux only inactivates CDKA. These suggest that Dap and Rux should be handled differently in computational studies.

### Robustness of cell cycles allows flexible orders of signaling events

Upon the consensus that biological signaling should be robust against errors and noises, many previous studies carefully checked if a model is sensitive to changes of initial conditions and parameter values^8^,^43^. However, ups and downs of a protein’s concentration can be caused by multiple regulators in complex ways, and not all parameter changes alter the occurrence and/or the order of signaling events. A more essential question may be to what extent cell cycle control can tolerate changes of signaling events.

Simulations of cell cycle phase compensation indicate that the increase of Dap and Wee changes some signaling events (Figs 3 and 4). As the equation of APCFzy shows, the timing of *Plx_Act_APCFzy* is determined by the parameter a_PlxFzy_:
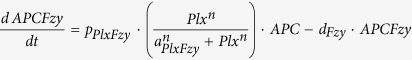


The smaller the parameter a_PlxFzy_ is, the earlier the event *Plx_Act_APCFzy* occurs. To examine to what extent the model is robust against changes of signaling events, we conducted simulations with parameters in Hill functions increased and decreased by 66.6%. In most cases cell cycles occurred (Table 3), indicating that the model is highly robust in the conventional sense. To ensure such results are not specific to this set of parameters, we identified two more sets of parameters that enable the model to generate all signaling events and repeated the simulations with parameters in Hill functions increased and decreased by 66.6% (Supplementary Table 3).

Upon results in Table 3 and Supplementary Table 3, events can be classified into three groups with respect to the alterations in their occurrence with perturbations to the system. The first are *critical events* that occur periodically with accurate timing. Many Skp2, E2F2, and CDKE events belong to this group, as the changes of parameters cause cell cycle fail. The opposite are *robust events* that occur robustly with considerably changed timing (most APCFzy and APCFzr events). Other events are in between, some cannot occur too early but can be late or absent, some cannot occur too late but can be present early, while all protein concentrations oscillate. Multiple statistical treatments of all events captured in three batches of simulations (Table 3, Supplementary Table 3, Supplementary Table 5) indicate that proteolysis events conducted by APCFzy and APCFzr and events conducted by CDKBa are significantly more robust than other events - their occurrence is hardly influenced by the increase and decrease of the controlling parameter. Such events, we posit, should be important. Bifurcation analysis also reveals that the model is sensitive to changes of E2F1 and E2F2 parameters (Supplementary Figure 7, 8).

To further examine the negative regulator Dap and Rux, we repeated all simulations described in Table 3 with the removal of Dap or Rux (s_Dap _= 0.0 or s_Rux _= 0.0) respectively. Compared with 14 failed cases (cell cycle fails) under all the parameter settings in Table 3, there were 10 failed cases when additionally s_Dap _= 0.0, and 17 failed cases when additionally s_Rux _= 0.0. This indicates that cell cycle progression is facilitated when the CDKE inhibitor Dap is absent, but is not when the CDKA inhibitor Rux is absent. Indeed, Rux mediates cells to enter into the S phase instead of entering into a new G1. The robustness of cell cycles against changes of signaling events and the classification of different events, for the first time, reveal a new dimension of cell cycle control. Similar to the conclusion from budding yeast^28^, these results suggest that, to a considerable extent, irreversibility of cell cycle phases is ensured by the robustness of timely destruction of cyclins and cyclin/Cdk complexes.

In simulations, when the parameter in some Hill functions is changed, it changes other events instead of, or together with, the defined one. For example, when a_FzrCycB_ is increased by even 50%, event 24, 25, 26, and 27 become absent, because delayed destruction of CycB allows more CDKBi and CDKBa to be formed. Simulations reveal that since the total amount of Cdk1 is a constant in the model, the binding of more CycB to Cdk1 hinders the binding of CycA to Cdk1. If the total amount of Cdk1 or the binding affinity of CycA/Cdk1 is increased, the absence of event 24, 25, 26, and 27 does not occur. Whether the delayed destruction of CycB by APCFzr influences CycA/Cdk1 binding is unreported and an interesting issue for experimental investigation.

## Discussion

Robustness as an essential property of cell cycle control has been examined by numerous studies, but its nature remains elusive. As experimental studies have uncovered considerable molecular interactions, intensive computational studies become not only necessary but also feasible. There are two challenges for one to build a computational model. The first is to select molecular interactions observed probably in more than one species; the second is to identify proper parameters to formalize molecular interactions. For the first, a model based on findings in one species has both pros and cons – data consistency is ensured, but assumptions are made due to data insufficiency. We think if conclusions do not ostensibly rely on detailed molecular interactions and molecular interactions are highly conserved, fewer assumptions on missing links in data and more findings from multiple species should be more preferable. In this study, the main conclusion - signaling events show flexible orders and key events show a robust order - should not sensitively rely on particular molecular interactions.

Multiple methods have been used in previous practices, including (1) to use numerical screening and specific sampling methods to explore the parameter space, (2) to explore constraints between parameters, (3) to tune a model to produce the wild-type phenotype, and (4) to prove that conclusions are independent of specific parameters. In this study we identify parameters not only upon the combined use of the above methods (except (1)), but also, for the first time, upon the generation of periodic signaling events. We find that many parameters enable the model to produce oscillating protein concentrations, but much fewer enable it to produce all signaling events. Based on the novel methods and results, the model reveals that robustness of cell cycle control determines the strict order of key events and allows flexible orders of other signaling events. Robust cell cycle control against changes of signaling events opens a new dimension of robust biological signaling. We do not claim that our analysis of signaling events is flawless, only that it may promote understanding the orders of signaling, in cell cycle control in particular and in multiple pathway interplay in general.

A question is, to what extent do the simulated events occur in cells? We point out that events are determined by protein concentrations that are computed using the widely adopted methods in previous studies, making periodic signaling events as convincing as oscillating protein concentrations. Further, in simulations if a protein’s concentration does not reach the defined threshold the defined event does not occur. Although this does not necessarily mean that the event would be absent *in vivo*, based on the well accepted mass-action rule, this stipulates that at least the event would occur for a shorter period *in vivo*, causing the order of related events likely changed. Thus, orders of events under *in vivo* situations can be reasonably examined by simulations.

The cell cycle control system comprises multiple and redundant components and feedbacks, among them are multiple Cdk inhibitors (e.g., Dap and Rux), regulators of feedbacks (e.g., Stg and Wee), and antagonistic E2F proteins (e.g., E2F1 and E2F4). In addition to the balance between Rb and E2F1, a balance between positive and negative E2Fs is also important^44^,^45^,^46^, and so far the roles of negative E2Fs and different Cdk inhibitors remain poorly understood. An important, but largely ignored, aspect of robust cell cycle control is the flexible phase compensation. Flexible lengths of cell cycle phases have been observed and examined in yeast^27^,^47^,^48^, but they are more important for and less understood in tissue and organ development in metazoans. As seen in *Drosophila* development, following prolonged G1/S or G2/M a cell produces shortened G2/M or G1/S to robustly maintain the total length of cell cycles^20^,^49^. Our model demonstrates how the CDKE-E2F1-CDKB long-range feedback, with the participation of inhibiting regulators such as Dap, Rux, and E2F2, realizes cell phase compensation and cell cycle arrest. For example, if E2F2 is absent, when Dap is low, the increased CDKE would mediate strong E2F1 degradation and cause escape from G1 arrest not to occur.

In cell cycles G1/S/G2/M phases are strictly ordered, but details remain controversial or unclear, including how the S phase and M phase are ordered^50^, how temporal ordering of mitotic exit events is achieved^14^,^51^, and to what extent signaling events should be ordered. Multiple mechanisms are proposed to explain ordered cell cycle progression^11^,^52^, including that the order of late mitotic events depends on the order in which different Cdk and APC substrates are dephosphorylated and destroyed^31^,^51^. The irreversibility of cell cycle progression is also argued not a consequence of protein degradation, instead, progression through the cell cycle is more like the cycle of a clothes washing machine and events must occur in a specific order^53^. Given that regulatory crosstalk between G1/S/G2/M phases widely exists^19^,^54^,^55^,^56^, it is important to explore to what extent signaling events are ordered. Our model demonstrates that many events can have flexible orders, or even be absent or persistently present. Since multiple feedbacks work in parallel and redundantly in cells, the flexible occurrence and orders of events are not a surprise, and successive events that maintain their relative orders under all conditions can be seen as “motifs” that indicate modularity of signaling.

Our simulations also reveal which events are more robust or critical than others. For example, to initiate a round of cell cycle CDKE activates E2F1, represses E2F2, and represses APCFzr. Thus, CDKE-related events are rather critical and sensitive to parameter changes. On the other hand, simulations under varied parameter settings reveal that the events of proteolysis of cyclins and Cdk complexes occur more robustly and are more strictly ordered than other events (Table 3, Supplementary Table 3, Supplementary Table 5). These results, together with the flexible coupling between the long-range CDKE-E2F1-CDKB feedback and the short-range Rb-E2F1 and Cdk1-Wee-Stg feedbacks under varied conditions, indicate that events in cells follow neither the domino nor the clock models^11^ because both of which stipulate a strict order, but agree better with the clothes washing machine hypothesis. We suggest that the flexibility and robustness of the cell cycle control can be more accurately metaphorized by a gear system - big and small gears, at different times and upon different regulatory signals, can be coupled in multiple ways to produce different events with flexible orders and to speed up or slow down specific cell cycle phases. The identification of more and more components participating in cell cycle regulation in a context-dependent manner strongly justifies this gear system model.

## Methods

### Formulate the model

The model uses 25 non-dimensionalized differential equations to describe interactions between 25 proteins and their complexes (Fig. 1; Supplementary Methods). Some technical details are as follows. First, unlike previous models^6^,^7^,^25^, upon the recent experimental findings^36^,^57^ we do not handle proteins’ multiple phosphorylation sites. Instead, proteins simply have active and inactive states (which correspond to all sites being either phosphorylated or dephosphorylated) and the transition between the two states is switch-like and controlled by the competition between the protein and its modifiers. Given that the Hill coefficient for Stg activation by Cdk1 is about 11^58^, coefficient = 6 is set in all Hill functions (except in the Rb auto-dephosphorylation process). Second, different methods have been used to describe nonlinearity of protein-protein interactions; we adopt the method that allows the modification of a protein to depend nonlinearly on the concentrations of its modifiers and linearly on the concentration of itself^11^. Third, since Cdks do not visibly fluctuate and overexpression of *cycE* can increase Cdk2 activity^20^, Cdks are at the maximal level but cyclins are at low levels. Fourth, we assume that different phosphorylation states do not affect a protein’s half-life.

### Parameterize the model

There are eight groups of parameters, including (1) s (synthesis rates), (2) d (decay rates), (3) u (SCF- or APC-mediated ubiquitination), (4) p (kinase- or phosphorylase-mediated phosphorylation or dephosphorylation), (5) k (binding), (6) kk (unbinding), (7) a (half maximal activation coefficient in Hill functions), and (8) r (half maximal repression coefficient in Hill functions) (Supplementary Methods). We first evaluated the ranges of parameters upon literature review, then explored the constraints between parameters, and finally determined the values of parameters by tuning the model to produce oscillating protein concentrations and periodic signaling event. We deliberately adopted a set of very simple initial conditions (Supplementary Table 4). Unrealistic though, they clearly indicate that the model does not demand specific initial conditions. Results in the main text are based on the set of parameters and the set of initial conditions.

To make analysis of events more reliable, we performed simulations with two more sets of parameters, and to facilitate bifurcation analysis we identified a second set of initial conditions. XPPAUT is used to examine the ranges of parameters.

### Solve the equations

Equations are solved under Linux using the second-order forward Runge-Kutta method with adaptive time steps controlled by two error thresholds. For a protein U described by 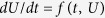, the error control should meet 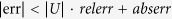. Here err = 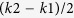, 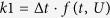, 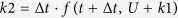, and *relerr *= *abserr* = 0.00001. In simulations, as long as 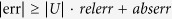 the time step is halved. The C code is available upon request.

### Capture signaling events

Using a programming tool we developed^59^, we define that, when the concentration of protein A reaches the half-maximal activation/repression coefficient in the Hill function describing how A nonlinearly activates/represses protein B, A sends the message *activation* or *repression* to B (the events are abbreviated as *A_Act_B* or *A_Rep_B*) (Fig. 1B). For example, when Skp2 > a_Skp2CycE_, Skp2 sends the message *ubiquitination* to CycE, and the message received in CycE is captured as the event *Skp2_Ubi_CycE*. In simulations all events are continuously captured in cells, and the windows showing signaling events and protein concentrations are captured using the program GIMP.

### Bifurcation analysis

With the original set of parameters and the new set of initial conditions, we used the program oscill8 to perform bifurcation analysis.

### Statistical analysis

Upon Table 3 and Supplementary Table 3, we quantify the model’s responses to changed timing of events by ranking 0/0 = 1, 0/1 = 2, 0/2 = 3, 1/1 = 4, 1/2 = 5, 2/2 = 6 (0, 1, and 2 indicate that protein concentrations do not oscillate, protein concentrations oscillate but some events do not occur periodically, and all events occur periodically). We then performed the global F test for all of the events, after which multiple comparisons (including Tamhane’s T2 multiple comparison test) were made.

## Additional Information

**How to cite this article**: Zhu, H. and Mao, Y. Robustness of cell cycle control and flexible orders of signaling events. *Sci. Rep.* **5**, 14627; doi: 10.1038/srep14627 (2015).

## Supplementary Material

Supplementary Information

## Figures and Tables

**Table 1:** G1/S and G2/M phase compensation.

Condition	G1/S	G2/M	G1/S~Total	G2/M~Total
Default parameters	8.03	3.07	72.34%	27.66%
s_Dap_=1.0→1.05	11.48	3.41	77.1%	22.9%
s_Wee_=1.0→1.8	5.7	2.55	69.1%	30.91%

**Table 2:** G1 arrest and escape of G1 arrest under different parameter values (empty blanks indicate default values).

d_Stg_	s_Dap_	s_Rux_	s_E2F2_	Results
	1.17			Arrested
		2.7		Arrested
			1.2	Arrested
0.3				Arrested
0.3	0.0			Escaped
0.3		0.0		Not escaped
0.3			0.0	Not escaped
0.5				Not arrested
0.5	1.1			Not arrested
0.5		2.0		Not arrested
0.5			1.1	Not arrested

**Table 3:** Signaling events and their influence on cell cycles.

Links	Interactions	References	Events	Parameters	Results ↗ ↘
1	CycE and Cdk2 form CDKE	16			
2	CDKE and Dap form CDKEDap	41			
3	CDKE mediates E2F1 degradation	20	CDKE_Ubi_E2F1	a_CDKEE2F1_	1/0
4	CDKE mediates Rux degradation	39	CDKE_Ubi_Rux	a_CDKERux_	1/1
5	CDKE turns Rb to Rbp	16	CDKE_Rep_Rb	a_CDKERb_	1/1
6	CDKE breaks RbE2F1 into Rbp and E2F1	16			
7	CDKE breaks DapE2F2 to Dap and E2F2	33,65	CDKE_Rep_DapE2F2	a_CDKEE2F2_	0/1
8	CDKE represses APCFzr	66,67	CDKE_Rep_APCFzr	r_CDKEFzr_	0/0
9	Skp2 degrades CycE	68	Skp2_Ubi_CycE	a_Skp2CycE_	1/1
10	Skp2 degrades CDKE	68	Skp2_Ubi_CDKE	a_Skp2CDKE_	1/0
11	Skp2 degrades Dap	69, 70, 71	Skp2_Ubi_Dap	a_Skp2Dap_	0/1
12	Rb and E2F1 form RbE2F1	25			
13	RbE2F1 dissociates into Rbp and E2F1	72			
14	E2F1 self-activation	37	E2F1_Act_E2F1	a_E2F1E2F1_	0/1
15	E2F1 activates CycE	16	E2F1_Act_CycE	a_E2F1CycE_	1/1
16	E2F1 activates CycA	16	E2F1_Act_CycA	a_E2F1CycA_	1/0
17	E2F1 activates Stg	20,73,74	E2F1_Act_Stgi	a_E2F1Stg_	1/1
18	E2F2 and Dap form DapE2F2	32,33			
19	DapE2F2 represses E2F1	33,46,75	DapE2F2_Rep_E2F1	r_DapE2F2E2F1_	1/0
20	DapE2F2 represses CycA	33,46,75	DapE2F2_Rep_CycA	r_DapE2F2CycA_	1/1
21	DapE2F2 represses CycB	33,46,75	DapE2F2_Rep_CycB	r_DapE2F2CycB_	1/1
22	CycA and Cdk1 form CDKA	16			
23	CDKA and Rux form CDKARux	40,76			
24	CDKA turns Stgi into Stga	77	CDKA_Act_Stga	a_CDKAStg_	0/1
25	CDKA represses Wee	77,78	CDKA_Rep_Wee	a_CDKAWee_	1/1
26	CDKA mediates E2F1 degradation	60,79	CDKA_Ubi_E2F1	a_CDKAE2F1_	1/0
27	CDKA represses APCFzr	66,67	CDKA_Rep_APCFzr	r_CDKAFzr_	1/0
28	CycB and Cdk1 forms CDKBi	16			
29	Wee turns CDKBa into CDKBi	16	Wee_Rep_CDKBa	a_WeeCDKB_	2/1
30	Stga turns CDKBi to CDKBa	16	Stga_Rep_CDKBi	a_StgCDKB_	2/1
31	CDKBa mediates E2F1 degradation	20	CDKBa_Ubi_E2F1	a_CDKBE2F1_	0/1
32	CDKBa turns Stgi to Stga	16	CDKBa_Act_Stga	a_CDKBStg_	2/2
33	CDKBa represses Wee	77,78	CDKBa_Rep_Wee	a_CDKBWee_	2/2
34	CDKBa activates Plx	43	CDKBa_Act_Plx	a_CDKBPlx_	2/2
35	CDKBa represses APCFzr	66,67	CDKBa_Rep_APCFzr	r_CDKBFzr_	2/1
36	Plx activates APCFzy generation	43	Plx_Act_APCFzy	a_PlxFzy_	1/2
37	APCFzy degrades CycA	80	APCFzy_Ubi_CycA	a_FzyCycA_	2/2
38	APCFzy degrades CDKA	80	APCFzy_Ubi_CDKA	a_FzyCDKA_	2/2
39	APCFzy degrades CycB	16,31	APCFzy_Ubi_CycB	a_FzyCycB_	2/2
40	APCFzy degrades CDKBi	31,81	APCFzy_Ubi_CDKBi	a_FzyCDKBi_	2/2
41	APCFzy degrades CDKBa	31,81	APCFzy_Ubi_CDKBa	a_FzyCDKBa_	2/2
42	APCFzr degrades CycA	80	APCFzr_Ubi_CycA	a_FzrCycA_	2/2
43	APCFzr degrades CDKA	80	APCFzr_Ubi_CDKA	a_FzrCDKA_	1/2
44	APCFzr degrades CycB	16,31	APCFzr_Ubi_CycB	a_FzrCycB_	1/2
45	APCFzr degrades CDKBi	31,81	APCFzr_Ubi_CDKBi	a_FzrCDKBi_	2/2
46	APCFzr degrades CDKBa	31,81	APCFzr_Ubi_CDKBa	a_FzrCDKBa_	2/2
47	APCFzr degrades Skp2	69,82,83	APCFzr_Ubi_Skp2	a_FzrSkp2_	1/0

Note: In the leftmost column, numbers indicating links between proteins (Fig. 1) also indicate signaling events. Association and dissociation between proteins are not modeled. In the rightmost column, ↗ and ↘ indicate 66.6% increase and decrease of the control parameter. 0: oscillating protein concentrations were not generated (cell cycle fails), 1: oscillations were generated with some events absent or present persistently, 2: oscillations were generated with all events occurring periodically.
