# Supplementary material for: Robustness of cell cycle control and flexible orders of signaling events
Source: Sci Rep. 2015 Sep 30;5:14627. doi: 10.1038/srep14627 (PMC4588580; doi:10.1038/srep14627)
Supplement: Supplementary Information [file srep14627-s1.pdf]

Robustness of cell cycle control and flexible orders of signaling events

Hao Zhu [1*], Yanlan Mao [2]

1. Bioinformatics Section, School of Basic Medical Sciences, Southern Medical University, Guangzhou, 510515, China.
2. MRC Laboratory for Molecular Cell Biology, University College London, Gower Street, London WC1E 6BT, UK

1 Supplementary Methods

(1) Non-dimensionalization of equations

The concentration of a protein [V] is determined by

$$\frac{d[V]}{dt} = synthesis - decay \pm transformations$$

Transformation includes phosphorylation and dephosphorylation (for ubiquitination, activation, or repression), association and dissociation. We use the following substitutions to non-dimensionalize and scale all equations by the level of APC

$$V \rightarrow V \cdot \frac{s_{APC}}{d_{APC}},$$

$$t \rightarrow t \cdot \frac{1}{d_{APC}},$$

$$s_i \rightarrow \frac{s_i}{s_{APC}}, d_i \rightarrow \frac{d_i}{d_{APC}}, u_i \rightarrow \frac{u_i}{d_{APC}}, p_i \rightarrow \frac{p_i}{d_{APC}}, kk_i \rightarrow \frac{kk_i}{d_{APC}}, k_i \rightarrow \frac{k_i s_{APC}}{d_{APC}^2}, c \rightarrow \frac{c \cdot d_{APC}}{s_{APC}}.$$

Thus, if [V] is nonlinearly transcriptionally activated by E2F1, bound and unbound by [W], nonlinearly phosphorylated (repression) by [X], nonlinearly ubiquitinated by APC, and has a decay contant, [V]'s equation is

$$\frac{d[V]}{d[t]} = s_{E2F1\_V} \cdot \left(\frac{[E2F1]^n}{a_{E2F1\_V}{}^n + [E2F1]^n} \right) - k_{VW} \cdot [V][W] + kk_{VW} \cdot [VW] - p_{X\_V} \cdot \left(\frac{[X]^n}{a_{X\_V}{}^n + [X]^n} \right)[V]$$
$$- u_{APC\_V} \cdot \left(\frac{[APC]^n}{a_{APC\_V}{}^n + [APC]^n} \right)[V] - d_V \cdot [V]$$

The non-dimensionalization and scaling yields

$$\frac{d\overline{V}}{d\overline{t}} = \overline{s_{E2F1\_V}} \cdot \left(\frac{\overline{E2F1}^n}{a_{E2F1\_V}{}^n + \overline{E2F1}^n} \right) - \overline{k_{VW}} \cdot VW + \overline{kk_{VW}} \cdot VW - \overline{p_{X\_V}} \cdot \left(\frac{\overline{X}^n}{a_{X\_V}{}^n + \overline{X}^n} \right) V$$
$$- \overline{u_{APC\_V}} \cdot \left(\frac{\overline{APC}^n}{a_{APC\_V}{}^n + \overline{APC}^n} \right) V - \overline{d_V} \cdot V$$

By assuming both the synthesis and decay rates of APC 1.0, non-dimensionalization and scaling facilitate parameter estimation. The s, k, kk, p, u, and d parameters indicate rates of

synthesis, association, dissociation, phosphorylation and dephosphorylation, ubiquitination, and decay. The a and r parameters in Hill functions indicate activation and repression.

(2) Equations with biological basis

The equations are formulated upon several assumptions, which are made based on experimental findings and widely adopted in previous studies. First, because cyclins bind to Cdks with high binding affinities and their dissociation half-lives are in the order of hours (Hochegger et al 2008), we do not handle dissociation and physial decay of cyclin/Cdk complexes. Instead, cyclin/Cdk complexes are destroyed by timely ubiquitination. Other proteins undergo a physical decay, and may also be ubiquinated if clear evidence exists. Second, because Cdks are presence in excess during the cell cycle (Reis et al 2004; Tyson et al 2008; Morgan 2007), we let the whole amount of Cdk1 and Cdk2 at the maximal level (Cdk1tot=Cdk2tot=1.0), and CycE/Cdk2, CycA/Cdk1, and CycB/Cdk1 binding depends on available free Cdk2 and Cdk1. Third, because competition between substrates, instead of the cooperation among multiple phosphorylation sites, makes main contribution to the ultrasensitive mutual protein phosphorylation and dephosphorylation (Kim and Ferrell 2007), we do not handle multiple phosphorylation states of Rb (Yang et al 2006; Haberichter et al 2007; Barik et al 2010), but simply let Rb have two states - active and inactive, and the transition between the two states is controlled by the competition between its modifiers. Fourth, similarly, we just consider CDKB's and Stg's active and inactive states (corresponds to all sites phosphorylated or dephosphorylated), and the transition between the two states is controlled by the competition between their modifiers (Wee, Stg, and active CDKB).

The CycE/Cdk2/Dap system

$$\begin{aligned}\frac{d\,CycE}{dt} = & \left(GF + \frac{E2F1^n}{a^n_{E2F1CycE} + E2F1^n}\right) \\ & - k_{CycECdk2}\cdot CycE\cdot\left(Cdk2\text{tot-}CDKE - CDKEDap\right) \\ & - u_{Skp2CycE}\cdot\left(\frac{Skp2^n}{a^n_{Skp2CycE} + Skp2^n}\right)\cdot CycE\end{aligned} \tag{1}$$

$$\begin{aligned}\frac{d\,CDKE}{dt} = & \, k_{CycECdk2}\cdot CycE\cdot\left(Cdk2tot\text{ - }CDKE - CDKEDap\right) \\ & - k_{CDKEDap}\cdot CDKE\cdot Dap \\ & + kk_{CDKEDap}\cdot CDKEDap \\ & - u_{Skp2CDKE}\cdot\left(\frac{Skp2^n}{a^n_{Skp2CDKE} + Skp2^n}\right)\cdot CDKE\end{aligned} \tag{2}$$

$$
\begin{aligned}
\frac{d\,Dap}{dt} = s_{Dap} & \\
& -k_{CDKEDap}\cdot CDKE\cdot Dap \\
& +kk_{CDKEDap}\cdot CDKEDap \\
& -k_{DapE2F2}\cdot Dap\cdot E2F2 \\
& +kk_{DapE2F2}\cdot DapE2F2 \\
& +p_{CDKEDapE2F2}\cdot\left(\frac{CDKE^n}{a^n_{CDKEDapE2F2}+CDKE^n}\right)\cdot DapE2F2 \\
& -u_{Skp2Dap}\cdot\left(\frac{Skp2^n}{a^n_{Skp2Dap}+Skp2^n}\right)\cdot Dap \\
& -d_{Dap}\cdot Dap
\end{aligned}
\qquad (3)
$$

$$
\begin{aligned}
\frac{d\,CDKEDap}{dt} = k_{CDKEDap}\cdot CDKE\cdot Dap & \\
-kk_{CDKEDap}\cdot CDKEDap &
\end{aligned}
\qquad (4)
$$

$$
\begin{aligned}
\frac{dSkp2}{dt} = s_{Skp2} & \\
& -u_{FzrSkp2}\cdot\left(\frac{APCFzr^n}{a^n_{APCFzrSkp2}+APCFzr^n}\right)\cdot Skp2 \\
& -d_{Skp2}\cdot Skp2
\end{aligned}
\qquad (5)
$$

GF (Growth factor) and E2F1 stimulate cyclin E expression independently (Yao et al 2008). Skp2, one of SCF (the name of the key ubiquitin-protein ligase including Skp1, cullin, and the F-box)'s main F-box proteins, is used to ubiquitinate G1/S targets (CycE and CDKE) (Moberg et al 2001). Decay of p21 and p27 is also through SCF-Skp2 (Amati and Vlach 1999; Guardavaccaro and Pagano 2006). In Drosophila, Skp2 interacts physically with Dap and targets Dap for ubiquitination (Dui et al 2013). Dap binds to CDKE to repress CDKE's activity (de Nooij et al 1996). Since in Drosophila eye *dap* is expressed widely ahead of the MF (morphogenetic furrow) (Baker 2007), *dap* is given a constant expression level. F-box proteins have basal expression level (Moberg et al 2001; see Gerard and Goldbeter 2009). Clear evidence indicates that at least in mammalian cells APC (the anaphase-promoting complex, the other main ubiquitin-protein ligase) degrades Skp2 in G1, which represents a principal mechanism by which APC maintains the G1 state (Bashir et al 2004; Wei et al 2004; Guardavaccaro and Pagano 2006).

Another recently identified master regulator of protein destruction in G1/S phases is CRL4Cdt2 (Haven and Walter 2011). In Drosophila, the relative roles of CRL4Cdt2 and Skp2 in the destruction of Dap remain poorly resolved, the destruction of E2Fs by CRL4Cdt2 may not occur in other species (Haven and Walter 2011), and how CRL4Cdt2 itself is regulated is unclear, except that it is quickly down-regulated after S phase (Zielke et al 2011; Haven and Walter 2011). In mammals, multiple pathways restrain E2F activities, and the function of

CRL4Cdt2 depends absolutely on the DNA-bound PCNA that is not explicitly described (Haven and Walter 2009). Due to these, we allowed interactions to be concise without including CRL4Cdt2 at this stage.

The Rb/E2F1/E2F2 system

$$\begin{aligned}\frac{d\,Rb}{dt} = {} & s_{Rb} \\ & -k_{RbE2F1}\cdot Rb\cdot E2F1 \\ & +kk_{RbE2F1}\cdot RbE2F1 \\ & +p_{dephoRbp}\cdot\left(\frac{Rbp^{1.0}}{K_{dephoRbp}^{1.0}+Rbp^{1.0}}\right) \\ & -p_{CDKERb}\cdot\left(\frac{CDKE^{n}}{a_{CDKERb}^{n}+CDKE^{n}}\right)\cdot Rb \\ & -d_{Rb}\cdot Rb\end{aligned} \tag{6}$$

$$\begin{aligned}\frac{d\,Rbp}{dt} = {} & p_{CDKERb}\cdot\left(\frac{CDKE^{n}}{a_{CDKERb}^{n}+CDKE^{n}}\right)\cdot Rb \\ & +p_{CDKERb}\cdot\left(\frac{CDKE^{n}}{a_{CDKERb}^{n}+CDKE^{n}}\right)\cdot RbE2F1 \\ & -p_{dephoRbp}\cdot\left(\frac{Rbp^{1.0}}{a_{dephoRbp}^{1.0}+Rbp^{1.0}}\right) \\ & -d_{Rbp}\cdot Rbp\end{aligned} \tag{7}$$

$$\begin{aligned}\frac{d\,E2F1}{dt} = {} & \left(\frac{s_{E2F1}+\dfrac{E2F1^{n}}{a_{E2F1E2F1}^{n}+E2F1^{n}}}{1+s_{E2F1}+\dfrac{E2F1^{n}}{a_{E2F1E2F1}^{n}+E2F1^{n}}}\cdot\frac{r_{DapE2F2E2F1}^{n}}{r_{DapE2F2E2F1}^{n}+DapE2F2^{n}}\right) \\ & -k_{RbE2F1}\cdot Rb\cdot E2F1 \\ & +kk_{RbE2F1}\cdot RbE2F1 \\ & +p_{CDKERb}\cdot\left(\frac{CDKE^{n}}{a_{CDKERb}^{n}+CDKE^{n}}\right)\cdot RbE2F1 \\ & -u_{CDKEE2F1}\cdot\left(\frac{CDKE^{n}}{a_{CDKEE2F1}^{n}+CDKE^{n}}\right)\cdot E2F1 \\ & -u_{CDKAE2F1}\cdot\left(\frac{CDKA^{n}}{a_{CDKAE2F1}^{n}+CDKA^{n}}\right)\cdot E2F1 \\ & -u_{CDKBaE2F1}\cdot\left(\frac{CDKBa^{n}}{a_{CDKBaE2F1}^{n}+CDKBa^{n}}\right)\cdot E2F1 \\ & -d_{E2F1}\cdot E2F1\end{aligned} \tag{8}$$

$$\frac{d\,E2F2}{dt} = s_{E2F2} - k_{DapE2F2} \cdot Dap \cdot E2F2 + kk_{DapE2F2} \cdot DapE2F2 + p_{CDKEDapE2F2} \cdot \left(\frac{CDKE^n}{a^n_{CDKEDapE2F2} + CDKE^n} \right) \cdot DapE2F2 - d_{E2F2} \cdot E2F2 \quad (9)$$

$$\frac{d\,DapE2F2}{dt} = k_{DapE2F2} \cdot Dap \cdot E2F2 - kk_{DapE2F2} \cdot DapE2F2 - p_{CDKEDapE2F2} \cdot \left(\frac{CDKE^n}{a^n_{CDKEDapE2F2} + CDKE^n} \right) \cdot DapE2F2 \quad (10)$$

$$\frac{d\,RbE2F1}{dt} = k_{RbE2F1} \cdot Rb \cdot E2F1 - kk_{RbE2F1} \cdot RbE2F1 - p_{CDKERb} \cdot \left(\frac{CDKE^n}{a^n_{CDKERb} + CDKE^n} \right) \cdot RbE2F1 \quad (11)$$

Rb is constantly expressed, binds to E2F1 (Haberichter et al 2007), is phosphorylated (inactivated) by CDKE, and is progressively dephosphorylated by PP1 and PP2A phosphatases (Kolupaeva and Janssens 2013). CDKE dissociates RbE2F1 to release active E2F1 and inactive Rbp. E2F1 activates its own transcription (Yao et al 2008). Binding by Rb protects E2F1 from ubiquitin-mediated degradation.

In mammals, unlike E2F1, the negative E2F protein E2F4 lacks a nuclear localization domain, is free to exit the nucleus, and needs p27/p130 (but not Rb) to co-locate to E2F-responsive promoters of target genes (Rayman et al 2002). p27 can bind to Cdk complexes by its N-terminal domain and to E2F4 by its C-terminal domain (Pippa et al 2012). At mid-late G1 when the expression of these genes is needed, the p27 (associated with p130/E2F4) would recruit Cdk complexes and these Cdk complexes (at late G1 cyclin D-Cdk4/6, and subsequently cyclin E-Cdk2) phosphorylate p130 and disrupt the p27/p130/E2F4 complexes (Malumbres and Barbacid, 2005; Pippa et al 2012). In *p130* double mutants, transcription of the *B-myb*, *cyclin A*, *cdc2 (cdk1)*, and *E2F1* genes is significantly derepressed in comparison with wild-type controls, but the *cyclin E* gene is not (Rayman et al 2002). Thus, p27 not only maintains cyclin E/cdk2 complexes inactive during early G1 to prevent premature entry into S phase, but also involves in the repression of genes necessary for DNA replication (Macaluso et al 2006; Plesca et al 2007; Pippa et al 2012). Upon these findings in mammalian cells (instead of the interaction between E2F2 and DP in Drosophila), in this model (E2F2 represents the negative E2F protein), we use Dap to represent p27 and p130, let DapE2F2 repress CycA, CycB, and E2F1, and let CDKE dissociate DapE2F2. Thus, CDKE activates E2F1

by breaking RbE2F1 and represses E2F2 by breaking DapE2F2.

It was suggested that after released from its association with Rb, E2F1 is phosphorylated by CDKE and CDKB (Reis and Edgar 2004) and CDKA (Xu et al 1994; Kitagawa et al 1995) for degradation. In Drosophila, it was later found that E2F1 is periodically destructed by CRL4Cdt2, and, by checking putative Cdk phosphorylation sites in E2F1, it was suggested that this destruction does not require CDKs (Shibutani et al 2008). How CRL4Cdt2 is quickly down-regulated after S phase is unclear (Ziekle et al 2011), and E2F1 destruction by CRL4Cdt2 may only happens in Drosophila (Haven and Walter 2011). More recently, it is reported that Cdt2 in CRL4Cdt2 is degraded by SCFFbxo11, and phosphorylation of Cdt2 by CDKs prevents Cdt2 from degradation by SCFFbxo11 (Rossi et al 2013). Therefore, CDKs' roles in preventing Cdt2 degradation may promote E2F1 destruction by CRL4Cdt2, in a way agreeing with the initial observation that phosphorylation of E2F1 by CDKs mediates E2F1 destruction. Considering situations in mammalian cells, we still let CDKs mediate E2F1 degradation, albeit possibly indirectly.

The CycA/Cdk1/Rux system

$$\begin{aligned}\frac{d\,CycA}{dt} &= \left(\frac{E2F1^n}{a^n_{E2F1CycA}+E2F1^n}\cdot\frac{r^n_{DapE2F2CycA}}{r^n_{DapE2F2CycA}+DapE2F2^n}\right)\\ &\quad -k_{CycACdk1}\cdot CycA\cdot\left(Cdk1tot-CDKBa-CDKBi-CDKA-CDKARux\right)\\ &\quad -u_{FzyCycA}\cdot\left(\frac{APCFzy^n}{a^n_{FzyCycA}+APCFzy^n}\right)\cdot CycA\\ &\quad -u_{FzrCycA}\cdot\left(\frac{APCFzr^n}{a^n_{FzrCycA}+APCFzr^n}\right)\cdot CycA\end{aligned} \tag{12}$$

$$\begin{aligned}\frac{d\,CDKA}{dt} &= k_{CycACdk1}\cdot CycA\cdot(Cdk1tot-CDKBa-CDKBi-CDKA-CDKARux)\\ &\quad -k_{CDKARux}\cdot CDKA\cdot Rux\\ &\quad +kk_{CDKARux}\cdot CDKARux\\ &\quad -u_{FzyCDKA}\cdot\left(\frac{APCFzy^n}{a^n_{FzyCDKA}+APCFzy^n}\right)\cdot CDKA\\ &\quad -u_{FzrCDKA}\cdot\left(\frac{APCFzr^n}{a^n_{FzrCDKA}+APCFzr^n}\right)\cdot CDKA\end{aligned} \tag{13}$$

$$
\begin{aligned}
\frac{d\,Rux}{dt} = \; & s_{Rux} \\
& -k_{CDKARux} \cdot CDKA \cdot Rux \\
& +kk_{CDKARux} \cdot CDKARux \\
& -u_{CDKERux} \cdot \left(\frac{CDKE^n}{a^n_{CDKERux} + CDKE^n} \right) \cdot Rux \\
& -d_{Rux} \cdot Rux
\end{aligned}
\tag{14}
$$

$$
\begin{aligned}
\frac{d\,CDKARux}{dt} = \; & k_{CDKARux} \cdot CDKA \cdot Rux \\
& - kk_{CDKARux} \cdot CDKARux
\end{aligned}
\tag{15}
$$

E2F1 transcriptionally activates CycA, which binds to Cdk1 to form CDKA. CycA and CDKA are ubiquitinated by APCFzr and APCFzy (Wolthuis et al 2008). Since *rux* is expressed widely in Drosophila eye disc (Escudero 2007), Rux has a constant expression level. Rux reversibly binds to and inhibits CDKA (Foley et al 1999; Avedisov et al 2000). CDKE down regulates Rux *in vivo* (Thomas et al 1997).

The CycB/Cdk1/Stg/Wee system

$$
\begin{aligned}
\frac{d\,CycB}{dt} = \; & \left(s_{CycB} + 0.4 \frac{r^n_{DapE2F2CycB}}{r^n_{DapE2F2CycB} + DapE2F2^n} \right) \\
& -k_{CycBCdk1} \cdot CycB \cdot (Cdk1tot - CDKBa - CDKBi - CDKA - CDKARux) \\
& -u_{FzyCycB} \cdot \left(\frac{APCFzy^n}{a^n_{FzyCycB} + APCFzy^n} \right) \cdot CycB \\
& -u_{FzrCycB} \cdot \left(\frac{APCFzr^n}{a^n_{FzrCycB} + APCFzr^n} \right) \cdot CycB
\end{aligned}
\tag{16}
$$

$$
\begin{aligned}
\frac{dCDKBi}{dt} = \; & k_{CycBCdk1} \cdot CycB \cdot (Cdk1tot - CDKBa - CDKBi - CDKA - CDKARux) \\
& + p_{WeeCDKB} \cdot \left(\frac{Wee^n}{a^n_{WeeCDKB} + Wee^n} \right) \cdot CDKBa \\
& - p_{StgaCDKB} \cdot \left(\frac{Stga^n}{a^n_{StgaCDKB} + Stga^n} \right) \cdot CDKBi \\
& -u_{FzyCDKBi} \cdot \left(\frac{APCFzy^n}{a^n_{FzyCDKBi} + APCFzy^n} \right) \cdot CDKBi \\
& -u_{FzrCDKBi} \cdot \left(\frac{APCFzr^n}{a^n_{FzrCDKBi} + APCFzr^n} \right) \cdot CDKBi
\end{aligned}
\tag{17}
$$

$$\frac{d\,CDKBa}{dt} = p_{StgaCDKB} \cdot \left(\frac{Stga^n}{a^n{}_{StgaCDKB} + Stga^n} \right) \cdot CDKBi$$
$$- p_{WeeCDKB} \cdot \left(\frac{Wee^n}{a^n_{WeeCDKB} + Wee^n} \right) \cdot CDKBa$$
$$- u_{FzyCDKBa} \left(\frac{APCFzy^n}{a^n_{FzyCDKBa} + APCFzy^n} \right) \cdot CDKBa \qquad (18)$$
$$- u_{FzrCDKBa} \cdot \left(\frac{APCFzr^n}{a^n_{FzrCDKBa} + APCFzr^n} \right) \cdot CDKBa$$

$$\frac{d\,Wee}{dt} = s_{Wee}$$
$$- p_{CDKAWee} \left(\frac{CDKA^n}{a^n_{CDKAWee} + CDKA^n} \right) \cdot Wee \qquad (19)$$
$$- p_{CDKBaWee} \cdot \left(\frac{CDKBa^n}{a^n_{CDKBaWee} + CDKBa^n} \right) \cdot Wee$$
$$- d_{Wee} \cdot Wee$$

$$\frac{d\,Stgi}{dt} = \left(\frac{s_{Stg} + \frac{E2F1^n}{a^n{}_{E2F1Stgi} + E2F1^n}}{1 + s_{Stg} + \frac{E2F1^n}{a^n{}_{E2F1Stgi} + E2F1^n}} \right)$$
$$- p_{CDKAStg} \cdot \left(\frac{CDKA^{3n}}{a^{3n}{}_{CDKAStg} + CDKA^{3n}} \right) \cdot Stgi$$
$$- p_{CDKBaStg} \cdot \left(\frac{CDKBa^n}{a^n{}_{CDKBaStg} + CDKBa^n} \right) \cdot Stgi \qquad (20)$$
$$- d_{Stgi} \cdot Stgi$$

$$\frac{d\,Stga}{dt} = p_{CDKAStg} \cdot \left(\frac{CDKA^{3n}}{a^{3n}_{CDKAStg} + CDKA^{3n}} \right) \cdot Stgi$$
$$+ p_{CDKBaStg} \cdot \left(\frac{CDKBa^n}{a^n_{CDKBaStg} + CDKBa^n} \right) \cdot Stgi \qquad (21)$$
$$- d_{Stga} \cdot Stga$$

As in previous models CycB has a constant expression level (Tsai et al 2008; Gerard and Goldbeter 2009), but its expression is also repressed by p130/E2F4 (Plesca et al 2007). CycA and CycB bind competitively to Cdk1. CDKB is inactivated by Wee, activated by the active Stg, and ubiquitinated by APCFzy and APCFzr (Morgan 2007). The loss of kinase activity of CDKB at the end of mitosis depends on the destruction of the cyclin subunits (Felix et al 1990). Wee has a constant expression level, and newly expressed Wee is active. The activities of Wee are

high during most of the cell cycle, but then decrease abruptly during mitosis after phosphorylation first by CDKA and then by CDKB (Watanabe et al 2005, Fung et al 2007). *stg* has a base level of expression (Edgar et al 1994), is also activated by E2F1 (Lehman et al 1999; Reis and Edgar 2004; Yao et al 2011), and newly expressed Stg is inactive. Stg is initially activated by CDKA (Fung et al 2007) and later by active CDKB.

The APC system

$$\frac{d\,Plx}{dt} = \left(\frac{CDKBa^n}{a^n_{CDKBPlx} + CDKBa^n}\right)\cdot\left(Plxtot - Plx\right) - d_{Plx}\cdot Plx \tag{22}$$

$$\frac{d\,APC}{dt} = s_{APC} - p_{PlxFzy}\cdot\left(\frac{Plx^n}{a^n_{PlxFzy} + Plx^n}\right)\cdot APC - p_{CDKFzr}\cdot\left(\frac{r^n_{CDKEFzr}}{r^n_{CDKEFzr} + CDKE^n}\cdot\frac{r^n_{CDKAFzr}}{r^n_{CDKAFzr} + CDKA^n}\cdot\frac{r^n_{CDKBaFzr}}{r^n_{CDKBaFzr} + CDKBa^n}\right)\cdot APC - d_{APC}\cdot APC \tag{23}$$

$$\frac{d\,APCFzy}{dt} = p_{PlxFzy}\cdot\left(\frac{Plx^n}{a^n_{PlxFzy} + Plx^n}\right)\cdot APC - d_{Fzy}\cdot APCFzy \tag{24}$$

$$\frac{d\,APCFzr}{dt} = p_{CDKFzr}\cdot\left(\frac{r^n_{CDKEFzr}}{r^n_{CDKEFzr} + CDKE^n}\cdot\frac{r^n_{CDKAFzr}}{r^n_{CDKAFzr} + CDKA^n}\cdot\frac{r^n_{CDKBaFzr}}{r^n_{CDKBaFzr} + CDKBa^n}\right)\cdot APC - d_{Fzr}\cdot APCFzr \tag{25}$$

Plx is needed for activation of APC by CDKB (Tsai et al 2008). CDKB turns Plx into the active form upon available inactive Plx, which is assumed at a high level (Tsai et al 2008). Plx activates the formation of APCFzy, and binding of APC to Fzy and Fzr (normally expressed in cellularized embryos, Raff et al 2002) consumes APC. As in previous models (Chen et al 2004; Novak et al 2004; Yang et al 2006), we assume that Fzy is always available for APC/Fzy binding, and that Fzr is always available for APC/Fzr binding whenever the binding is not periodically inhibited by CDKE, CDKA, and CDKB (Zielke et al 2008; Narbonne-Reveau et al 2008).

(3) Parameters with biological basis

Supplementary Table 1 Parameter values

Param	Description	Val	Note	Param	Description	Val	Note
n	Hill functions' coefficient	6.0	1	$u_{Skp2Dap}$	Skp2 ubiquitinates Dap	2.0	11
s_{APC}	APC synthesis rate	1.0	2	$u_{Skp2CycE}$	Skp2 ubiquitinates CycE	2.0	
d_{APC}	APC decay rate	1.0		$u_{Skp2CDKE}$	Skp2 ubiquitinates CDKE	2.0	
Cdk1tot	Total amount of Cdk1	1.0	3	$u_{FzrSkp2}$	Fzr ubiquitinates Skp2	1.0	12
Cdk2tot	Total amount of Cdk2	1.0		$u_{FzyCycA}$	Fzy ubiquitinates CycA	1.0	
Plxtot	Total amount of Plx	1.0		$u_{FzyCDKA}$	Fzy ubiquitinates CDKA	0.5	
GF	Growth factor for CycE	0.5		$u_{FzyCycB}$	Fzy ubiquitinates CycB	1.5	
s_{E2F1}	E2F1 synthesis rate	0.2	4	$u_{FzyCDKBi}$	Fzy ubiquitinates CDKBi	0.5	
s_{Stgi}	String synthesis rate	0.2		$u_{FzyCDKBa}$	Fzy ubiquitinates CDKBa	0.5	
s_{CycB}	CycB synthesis rate	0.2		$u_{FzrCycA}$	Fzr ubiquitinates CycA	1.5	
s_{E2F2}	E2F2 synthesis rate	1.0	5	$u_{FzrCDKA}$	Fzr ubiquitinates CDKA	0.15	
s_{Dap}	Dap synthesis rate	1.0		$u_{FzrCycB}$	Fzr ubiquitinates CycB	1.0	
s_{Rux}	Rux synthesis rate	1.0		$u_{FzrCDKBi}$	Fzr ubiquitinates CDKBi	0.1	
s_{Skp2}	Skp2 synthesis rate	1.0		$u_{FzrCDKBa}$	Fzr ubiquitinates CDKBa	0.1	
s_{Rb}	Rb synthesis rate	1.0		$u_{CDKERux}$	CDKE degrades Rux	1.0	13
s_{Wee}	Wee synthesis rate	1.0		$u_{CDKEE2F1}$	CDKE degrades E2F1	2.0	
d_{Rux}	Rux decay rate	1.0	6	$u_{CDKAE2F1}$	CDKA degrades E2F1	1.0	
d_{Wee}	Wee decay rate	1.0		$u_{CDKBaE2F1}$	CDKBa degrades E2F1	2.0	
d_{Plx}	Plx decay rate	1.0		$a_{Skp2CDKE}$	Skp2 ubiquitinates CDKE	0.55	14
d_{Stgi}	Inactive Stg decay rate	1.0		$a_{Skp2CycE}$	Skp2 ubiquitinates CycE	0.55	
d_{Stga}	Active Stg decay rate	1.0		$a_{Skp2Dap}$	Skp2 ubiquitinates Dap	0.7	
d_{Dap}	Dap decay rate	1.0		$a_{E2F1CycE}$	E2F1 activates CycE	0.28	15
d_{Fzy}	APCFzy decay rate	1.0		$a_{E2F1CycA}$	E2F1 activates CycA	0.28	
d_{Fzr}	APCFzr decay rate	1.0		$a_{E2F1E2F1}$	E2F1 activates E2F1	0.205	
d_{Skp2}	Skp2 decay rate	1.0		$a_{E2F1Stgi}$	E2F1 activates Stg	0.28	
d_{Rb}	Rb decay rate	2.0		$a_{FzrSkp2}$	Fzr ubiquitinates Skp2	0.2	16
d_{Rbp}	Rbp decay rate	1.0		$a_{FzyCycA}$	Fzy ubiquitinates CycA	0.07	
d_{E2F1}	E2F1 decay rate	0.1		$a_{FzyCDKA}$	Fzy ubiquitinates CDKA	0.1	
d_{E2F2}	E2F2 decay rate	2.5		$a_{FzyCycB}$	Fzy ubiquitinates CycB	0.07	
$k_{CycECdk2}$	CycE/Cdk2 binding	2.0	7	$a_{FzyCDKBi}$	Fzy ubiquitinates CDKBi	0.1	
$k_{CycACdk1}$	CycA/Cdk1 binding	2.0		$a_{FzyCDKBa}$	Fzy ubiquitinates CDKBa	0.1	
$k_{CycBCdk1}$	CycB/Cdk1 binding	2.0		$a_{FzrCycA}$	Fzr ubiquitinates CycA	0.15	
k_{RbE2F}	Rb/E2F1 binding	5.0		$a_{FzrCDKA}$	Fzr ubiquitinates CDKA	0.25	
$k_{CDKARux}$	CDKA/Rux binding	1.5		$a_{FzrCycB}$	Fzr ubiquitinates CycB	0.05	
$k_{CDKEDap}$	CDKE/Dap binding	1.5		$a_{FzrCDKBi}$	Fzr ubiquitinates CDKBi	0.15	
$k_{DapE2F2}$	Dap/E2F2 binding	3.0		$a_{FzrCDKBa}$	Fzr ubiquitinates CDKBa	0.15	
$kk_{CDKARux}$	CDKARux unbinding	1.0	8	a_{CDKERb}	CDKE phosphorylates	0.3	17
$kk_{CDKEDap}$	CDKEDap unbinding	1.0		$a_{CDKERux}$	CDKE degrades Rux	0.3	
kk_{RbE2F}	RbE2F1 unbinding	1.0		$a_{CDKEE2F1}$	CKDE degrades E2F1	0.3	
$kk_{DapE2F2}$	DapE2F2 unbinding	1.0		$r_{CDKEFzr}$	CDKE represses Fzr	0.25	
$p_{WeeCDKBa}$	Wee represses CDKB	1.0	9	$a_{CDKEDapE2F2}$	CDKE represses DapE2F2	0.2	
$p_{StgaCDKBa}$	Stga activates CDKB	1.0		$r_{CDKAFzr}$	CDKA represses Fzr	0.14	18
$p_{CDKAWee}$	CDKA inactivates Wee	1.0		$a_{CDKAWee}$	CDKA represses Wee	0.14	
$p_{CDKBaWee}$	CDKBa inactivates Wee	1.0		$a_{CDKAStgi}$	CDKA activates Stg	0.14	
p_{PlxFzy}	Plx activates APCFzy	1.0		$a_{CDKAE2F1}$	CDKA degrades E2F1	0.14	
p_{CDKFzr}	CDKs inactivates APCFzr	1.0		$a_{CDKBaPlx}$	CDKBa activates Plx	0.2	19
p_{CDKERb}	CDKE inactivates Rb	3.0		$r_{CDKBaFzr}$	CDKB represses Fzr	0.18	
$p_{CDKE\_DapE2F2}$	CDKE inactivates DapE2F2	5.0		$a_{CDKBaStgi}$	CDKB activates Stg	0.18	
$p_{CDKAStgi}$	CDKA activates Stg	2.0		$a_{CDKBaWee}$	CDKB represses Wee	0.18	
$p_{CDKBaStgi}$	CDKB activates Stg	1.0		$a_{CDKBaE2F}$	CDKB degrades E2F1	0.24	
$p_{dephoRbp}$	Rb auto-dephosphorylation	1.0		$a_{StgaCDKBi}$	Stg activates CDKB	0.155	20
$r_{DapE2F2E2F1}$	DapE2F2 represses E2F1	0.2	10	$a_{WeeCDKBa}$	Wee represses CDKB	0.5	
$r_{Dap2E2F2CycA}$	DapE2F2 represses CycA	0.2		a_{PlxFzy}	Plx activates Fzy	0.4	21
$r_{Dap2E2F2CycB}$	DapE2F2 represses CycB	0.2		$K_{dephoRbp}$	Rb auto-dephosphorylation	0.1	22

1. Protein interactions in feedbacks (especially, the activation of Stg by Cdk1 and Cdk1-induced degradation of CycA and CycB) are highly ultrasensitive and switch-like (Yang

and Ferrell 2013). The apparent Hill coefficient for Stg activation by Cdk1 is 11 (Trunnell et al 2011). We choose a moderate value for all nonlinear processes. 2. We scale variables by the syhtnesis and decay levels of APC. 3. Cdk2, Cdk1, and Plx have the same total amount. 4. Since the expression of E2F1, Stg, and CycB is also regulated by E2F1 and E2F2, these contant syhthesis rates should be relatively small to allow the regulation to play significant roles. 5. These proteins are assumed to be expressed at the high levels, and these settings allow these parameters to be removed. 6. These settings allow some parameters to be removed. Because E2F1's level is dynamically degraded by multiple proteins, its decay rate should be small. In contrast, for E2F2, which is not targeted for degradation by other proteins, a large decay rate is necessary. 7-8. Binding rates should be larger than unbinding rates, and these settings allow unbinding rates to be removed. 9. These settings allow some parameters to be removed, to let the nonlinear processes controlled mainly by the thresholds in the Hill functions. 10. Thresholds in negative Hill functions should be small. 11. Skp2 should ubiquitenates G1 cyclins and Cdks timely. 12. These parameters are tuned based on the levels of CycA, CDKA, CycB, CDKBi, and CDKBa. 13. The CDKE and CDKB mediated degradation of E2F1 should be strong to allow cell cycle phase compensation to occur. Parameters beginning with a and r are coefficients in Hill functions controlling non-linearity of protein interactions. 14. We assume that Skp2 downgrades CycE and CDKE at the same time, but downgrades Dap slightly later. 15. Tuning of the model indicates that the self-activation of E2F1 should occur earlier than the activation of CycE, CycA, and Stg. 16. Ubiquitination of CycA/CDKA/CycB/CDKB by APCFzy occurs earlier than ubiquitination of these proteins by APCFzr. 17. Tuning of the model indicates that degradation of DapE2F2 should occur early. 18-19. These parameters are tuned. 20. Normally CDKB is repressed by the high level of Wee. To enable slightly increased active Stg to gradually turn CDKBi into CDKBa, the threshold for Stg activation of CDKB should be small. 21. The value produces a proper delay for APC binding to Fzy. 22. Rb always undergoes a slow auto-dephosphorylation process; in this Hill function the coefficient n=1.0.

2 More about parameters and initial conditions

Upon the original parameters (Supplementary Table 1) and the new initial conditions (Supplementary Table 4), we used the program XPPAUT to identify each parameter's range (other parameters remained unchanged) that makes the model generate oscillating protein concentrations (Supplementary Table 2). Most parameters have a very large range, indicating the robustness of the model. Some parameters of E2F2, Skp2, E2F1, and CDKE have a narrow range. For example, s_{E2F2}=1.0-1.1, $r_{DapE2F2E2F1}$=0.18-0.2, s_{Skp2}=0.8-1.0, $a_{Skp2CDKE}$=0.55, $a_{E2F1E2F1}$=0.2-0.28, $a_{CDKEE2F1}$=0.3, $r_{CDKEFzr}$=0.25, and $a_{CDKEDapE2F2}$=0.2. These narrow ranges indicate that the model is sensitive to changes of these parameters. A large Hill coefficient is not a must for the model, and in all equations *n* can be as small as 3.

We deliberately adopted a set of very simple initial conditions (Supplementary Table 4). Unrealistic though, they indicate that the model does not demand specific initial conditions. These initial conditions do not allow the program XPPAUT to identify a fixed point for bifurcation analysis. We identified another set of initial conditions that are biologically more reasonable and allow XPPAUT to identify a fixed point of the model (Supplementary Table 4).

To reliably analyze the robustness of the model against changes of parameters and of events, we carefully explored constraints among parameters and identified two more sets of parameters that make the model generate both oscillating protein concentrations and all signaling events (Supplementary Table 3). Notice that, while many parameter values make the model generate oscillating protein concentrations, very few make the model generate all signaling events. This indicates that the three sets of parameters are biologically more reasonably constrained. We find that to generate all signaling events, significant changes of some half-maximal activating and inhibiting coefficients in Hill functions are not allowed, this explains why considerable half-maximal activating and inhibiting coefficients in the second and third set of parameters do not differ much.

Supplementary Table 2 Ranges of parameters
(under the new initial conditions in Supplementary Table 4)

Parameter	Value	Range	Parameter	Value	Range
n	6	3-10∈[0-10]	$u_{FzrSkp2}$	1.0	0.6-1.1∈[0-3]
Cdk1tot	1.0	0.8.2.0∈[0-2]	$u_{FzyCycA}$	1.0	0.1-1.0,1.1-1.3∈[0-3]
Cdk2tot	1.0	0.7-2.0∈[0-2]	$u_{FzyCDKA}$	0.5	0.0-3.0∈[0-3]
Plxtot	1.0	0.6-2.0∈[0-2]	$u_{FzyCycB}$	1.5	0.0-3.0∈[0-3]
GF	0.5	0.3-1.6∈[0-2]	$u_{FzyCDKBi}$	0.5	0.0-3.0∈[0-3]
s_{E2F1}	0.2	0.2-1.5∈[0-2]	$u_{FzyCDKBa}$	0.5	0.0-1.3∈[0-3]
s_{Stgi}	0.2	0.0-2.0∈[0-2]	$u_{FzrCycA}$	1.5	0.0-1.5,1.7-1.8∈[0-3]
s_{CycB}	0.2	0.0-2.0∈[0-2]	$u_{FzrCDKA}$	0.15	0.0-0.1,0.3-0.6∈[0-3]
s_{E2F2}	1.0	1.0-1.1∈[0-2]	$u_{FzrCycB}$	1.0	0.0-3.0∈[0-3]
s_{Dap}	1.0	0.0-1.1∈[0-2]	$u_{FzrCDKBi}$	0.1	0.1-3.0∈[0-3]
s_{Rux}	1.0	0.0-2.0∈[0-2]	$u_{FzrCDKBa}$	0.1	0.0-3.0∈[0-3]
s_{Skp2}	1.0	0.8-1.0∈[0-2]	$u_{CDKERux}$	1.0	0.0-0.5,0.9-3.0∈[0-3]
s_{Rb}	1.0	1.0-2.0∈[0-2]	$u_{CDKEE2F1}$	2.0	2.0-2.9∈[0-3]
s_{Wee}	1.0	0.0-1.3∈[0-2]	$u_{CDKAE2F1}$	1.0	1.0-2.9∈[0-3]
d_{Rux}	1.0	0.3-3.0∈[0-3]	$u_{CDKBaE2F1}$	2.0	2.0-3.0∈[0-3]
d_{Wee}	1.0	0.4-3.0∈[0-3]	$a_{Skp2CDKE}$	0.55	0.55,0.8-1.0∈[0-1]
d_{Plx}	1.0	0.4-2.8∈[0-3]	$a_{Skp2CycE}$	0.55	0.0-0.55∈[0-1]
d_{Stgi}	1.0	0.0-3.0∈[0-3]	$a_{Skp2Dap}$	0.7	0.7-0.8∈[0-1]
d_{Stga}	1.0	0.3-2.2∈[0-3]	$a_{E2F1CycE}$	0.28	0.1-1.0∈[0-1]
d_{Dap}	1.0	0.0-1.0∈[0-5]	$a_{E2F1CycA}$	0.28	0.2-0.55∈[0-1]
d_{Fzy}	1.0	0.3-3.0∈[0-3]	$a_{E2F1E2F1}$	0.205	0.2-0.28∈[0-1]
d_{Fzr}	1.0	0.9-2.0∈[0-3]	$a_{E2F1Stgi}$	0.28	0.0-0.5∈[0-1]
d_{Skp2}	1.0	1.0-1.25∈[0-3]	$a_{FzrSkp2}$	0.2	0.2-0.4∈[0-1]
d_{Rb}	2.0	0.0-2.0∈[0-3]	$a_{FzyCycA}$	0.07	0.07-0.5∈[0-1]
d_{Rbp}	1.0	0.0-1.25∈[0-3]	$a_{FzyCDKA}$	0.1	0.05-1.0∈[0-1]
d_{E2F1}	0.1	0.1-0.4∈[0-3]	$a_{FzyCycB}$	0.07	0.0-1.0∈[0-1]
d_{E2F2}	2.5	2.2-2.5∈[0-3]	$a_{FzyCDKBi}$	0.1	0.0-1.0∈[0-1]
$k_{CycECdk2}$	2.0	0.8-10.0∈[0-10]	$a_{FzyCDKBa}$	0.1	0.05-1.0∈[0-1]
$k_{CycACdk1}$	2.0	0.2-10.0∈[0-10]	$a_{FzrCycA}$	0.15	0.15-1.0∈[0-1]
$k_{CycBCdk1}$	2.0	0.4-10.0∈[0-10]	$a_{FzrCDKA}$	0.25	0.0-0.1,0.2-1.0∈[0-1]
k_{RbE2F}	5.0	4.8-10.0∈[0-10]	$a_{FzrCycB}$	0.05	0.0-1.0∈[0-1]
$k_{CDKARux}$	1.5	0.5-3.5∈[0-10]	$a_{FzrCDKBi}$	0.15	0.0-0.25∈[0-1]
$k_{CDKEDap}$	1.5	1.5-3.5∈[0-10]	$a_{FzrCDKBa}$	0.15	0.0-1.0∈[0-1]
$k_{DapE2F2}$	3.0	3.0-3.4∈[0-10]	a_{CDKERb}	0.3	0.3-1.0∈[0-1]
$kk_{CDKARux}$	1.0	0.5-5.0∈[0-5]	$a_{CDKERux}$	0.3	0.0-1.0∈[0-1]
$kk_{CDKEDap}$	1.0	0.5-1.0∈[0-5]	$a_{CDKEE2F1}$	0.3	0.3∈[0-1]
kk_{RbE2F}	1.0	0.0-1.0∈[0-5]	$r_{CDKEFzr}$	0.25	0.2-0.3∈[0-1]
$kk_{DapE2F2}$	1.0	0.5-1.0∈[0-5]	$a_{CDKEDapE2F2}$	0.2	0.2∈[0-1]
$p_{WeeCDKBa}$	1.0	0.0-3.0∈[0-3]	$r_{CDKAFzr}$	0.14	0.1-1.0∈[0-1]
$p_{StgaCDKBa}$	1.0	0.4-3.0∈[0-3]	$a_{CDKAWee}$	0.14	0.0-0.2∈[0-1]
$p_{CDKAWee}$	1.0	0.2-3.0∈[0-3]	$a_{CDKAStgi}$	0.14	0.0-0.15∈[0-1]
$p_{CDKBaWee}$	1.0	0.1-3.0∈[0-3]	$a_{CDKAE2F1}$	0.14	0.1-0.14∈[0-1]
p_{PlxFzy}	1.0	0.1-3.0∈[0-3]	$a_{CDKBaPlx}$	0.2	0.15-0.35∈[0-1]
p_{CDKFzr}	1.0	0.4-1.4,2.0-3.0∈[0-3]	$r_{CDKBaFzr}$	0.18	0.05-0.15,0.3-1.0∈[0-1]
p_{CDKERb}	3.0	0.0-3.0∈[0-3]	$a_{CDKBaStgi}$	0.18	0.0-1.0∈[0-1]
$p_{CDKEDapE2F2}$	5.0	4.2-5.0∈[2.5-7.5]	$a_{CDKBaWee}$	0.18	0.0-0.35∈[0-1]
$p_{CDKAStgi}$	2.0	0.4-3.0∈[0-3]	$a_{CDKBaE2F}$	0.24	0.1-0.24∈[0-1]
$p_{CDKBaStgi}$	1.0	0.0-3.0∈[0-3]	$a_{StgaCDKBi}$	0.155	0.05-0.3∈[0-1]
$p_{dephoRbp}$	1.0	1.0-3.0∈[0-3]	$a_{WeeCDKBa}$	0.5	0.4-1.0∈[0-1]
$K_{dephoRbp}$	0.1	0.0-0.1∈[0-1]	a_{PlxFzy}	0.4	0.2-0.75∈[0-1]
$u_{Skp2Dap}$	2.0	0.9-2.0∈[0-3]	$r_{DapE2F2E2F1}$	0.2	0.18-0.2∈[0-1]
$u_{Skp2CycE}$	2.0	0.0-1.3,1.8-3.0∈[0-3]	$r_{Dap2E2F2CycA}$	0.2	0.1-1.0∈[0-1]
$u_{Skp2CDKE}$	2.0	0.7-1.7,2.0-3.0∈[0-3]	$r_{Dap2E2F2CycB}$	0.2	0.0-1.0∈[0-1]

Supplementary Table 3 The second and third sets of parameters
(↗ and ↘ indicate 66.6% increase and decrease of the control parameter)

Set two	Value	Set two	Value	Results ↗ ↘	Set three	Value	Set three	Value	Results ↗ ↘
n	6	$u_{FzrSkp2}$	1.0		n	6	$u_{FzrSkp2}$	1.0	
Cdk1tot	1.0	$u_{FzyCycA}$	1.0		Cdk1tot	1.0	$u_{FzyCycA}$	1.0	
Cdk2tot	1.0	$u_{FzyCDKA}$	1.0		Cdk2tot	1.0	$u_{FzyCDKA}$	1.0	
Plxtot	1.0	$u_{FzyCycB}$	1.0		Plxtot	1.0	$u_{FzyCycB}$	1.0	
GF	0.6	$u_{FzyCDKBi}$	1.0		GF	0.6	$u_{FzyCDKBi}$	1.0	
s_{E2F1}	1.8	$u_{FzyCDKBa}$	1.0		s_{E2F1}	1.8	$u_{FzyCDKBa}$	1.0	
s_{Stgi}	0.8	$u_{FzrCycA}$	1.5		s_{Stgi}	0.8	$u_{FzrCycA}$	1.5	
s_{CycB}	0.8	$u_{FzrCDKA}$	0.4		s_{CycB}	0.8	$u_{FzrCDKA}$	0.4	
s_{E2F2}	0.8	$u_{FzrCycB}$	1.5		s_{E2F2}	0.8	$u_{FzrCycB}$	1.5	
s_{Dap}	0.8	$u_{FzrCDKBi}$	1.1		s_{Dap}	0.8	$u_{FzrCDKBi}$	1.1	
s_{Rux}	0.8	$u_{FzrCDKBa}$	1.1		s_{Rux}	0.8	$u_{FzrCDKBa}$	1.1	
s_{Skp2}	0.8	$u_{CDKERux}$	1.5		s_{Skp2}	0.8	$u_{CDKERux}$	1.5	
s_{Rb}	0.8	$u_{CDKEE2F1}$	2.2		s_{Rb}	0.8	$u_{CDKEE2F1}$	2.2	
s_{Wee}	1.0	$u_{CDKAE2F1}$	2.2		s_{Wee}	1.0	$u_{CDKAE2F1}$	2.2	
d_{Rux}	1.5	$u_{CDKBaE2F1}$	2.2		d_{Rux}	1.5	$u_{CDKBaE2F1}$	2.2	
d_{Wee}	1.5	$a_{Skp2CDKE}$	0.6	0/0	d_{Wee}	1.5	$a_{Skp2CDKE}$	0.6	0/0
d_{Plx}	1.5	$a_{Skp2CycE}$	0.6	0/1	d_{Plx}		$a_{Skp2CycE}$	0.6	1/1
d_{Stgi}	1.5	$a_{Skp2Dap}$	0.7	1/0	d_{Stgi}	1.5	$a_{Skp2Dap}$	0.7	1/1
d_{Stga}	1.5	$a_{E2F1CycE}$	0.28	1/0	d_{Stga}	1.5	$a_{E2F1CycE}$	0.28	2/0
d_{Dap}	0.8	$a_{E2F1CycA}$	0.28	2/1	d_{Dap}	0.8	$a_{E2F1CycA}$	0.28	2/1
d_{Fzy}	1.5	$a_{E2F1E2F1}$	0.205	2/1	d_{Fzy}	1.5	$a_{E2F1E2F1}$	0.205	2/1
d_{Fzr}	1.0	$a_{E2F1Stgi}$	0.28	1/1	d_{Fzr}	1.0	$a_{E2F1Stgi}$	0.28	1/2
d_{Skp2}	1.1	$a_{FzrSkp2}$	0.15	2/0	d_{Skp2}	1.1	$a_{FzrSkp2}$	0.2	1/1
d_{Rb}	1.0	$a_{FzyCycA}$	0.07	2/2	d_{Rb}	1.0	$a_{FzyCycA}$	0.07	2/2
d_{Rbp}	1.1	$a_{FzyCDKA}$	0.1	2/2	d_{Rbp}	1.1	$a_{FzyCDKA}$	0.1	2/2
d_{E2F1}	0.05	$a_{FzyCycB}$	0.07	2/2	d_{E2F1}	0.05	$a_{FzyCycB}$	0.07	2/2
d_{E2F2}	2.4	$a_{FzyCDKBi}$	0.1	2/2	d_{E2F2}	2.4	$a_{FzyCDKBi}$	0.1	2/2
$k_{CycECdk2}$	6.0	$a_{FzyCDKBa}$	0.1	2/2	$k_{CycECdk2}$	6.0	$a_{FzyCDKBa}$	0.1	2/2
$k_{CycACdk1}$	3.0	$a_{FzrCycA}$	0.15	2/2	$k_{CycACdk1}$	3.0	$a_{FzrCycA}$	0.15	2/2
$k_{CycBCdk1}$	3.0	$a_{FzrCDKA}$	0.2	2/2	$k_{CycBCdk1}$	3.0	$a_{FzrCDKA}$	0.2	2/2
k_{RbE2F}	2.0	$a_{FzrCycB}$	0.15	2/2	k_{RbE2F}	2.0	$a_{FzrCycB}$	0.15	2/2
$k_{CDKARux}$	2.0	$a_{FzrCDKBi}$	0.15	2/2	$k_{CDKARux}$	2.0	$a_{FzrCDKBi}$	0.15	2/2
$k_{CDKEDap}$	1.0	$a_{FzrCDKBa}$	0.15	2/2	$k_{CDKEDap}$	1.0	$a_{FzrCDKBa}$	0.15	2/2
$k_{DapE2F2}$	3.2	a_{CDKERb}	0.35	2/1	$k_{DapE2F2}$	3.2	a_{CDKERb}	0.35	2/1
$kk_{CDKARux}$	2.0	$a_{CDKERux}$	0.35	2/1	$kk_{CDKARux}$	2.0	$a_{CDKERux}$	0.35	2/1
$kk_{CDKEDap}$	2.0	$a_{CDKEE2F1}$	0.65	1/1	$kk_{CDKEDap}$	2.0	$a_{CDKEE2F1}$		1/0
kk_{RbE2F}	0.8	$r_{CDKEFzr}$	0.3	2/0	kk_{RbE2F}	0.8	$r_{CDKEFzr}$	0.3	2/0
$kk_{DapE2F2}$	0.8	$a_{CDKEDapE2F2}$	0.25	1/0	$kk_{DapE2F2}$	0.8	$a_{CDKEDapE2F2}$	0.25	1/1
$p_{WeeCDKBa}$	2.0	$r_{CDKAFzr}$	0.14	2/2	$p_{WeeCDKBa}$	2.0	$r_{CDKAFzr}$	0.14	2/2
$p_{StgaCDKBa}$	2.0	$a_{CDKAWee}$	0.14	2/1	$p_{StgaCDKBa}$	2.0	$a_{CDKAWee}$	0.14	2/2
$p_{CDKAWee}$	2.0	$a_{CDKAStgi}$	0.14	2/2	$p_{CDKAWee}$	2.0	$a_{CDKAStgi}$	0.1	2/2
$p_{CDKBaWee}$	2.0	$a_{CDKAE2F1}$	0.14	0/2	$p_{CDKBaWee}$	2.0	$a_{CDKAE2F1}$	0.12	2/1
p_{PlxFzy}	2.0	$a_{CDKBaPlx}$	0.2	2/0	p_{PlxFzy}	2.0	$a_{CDKBaPlx}$	0.2	2/2
p_{CDKFzr}	3.0	$r_{CDKBaFzr}$	0.18	2/2	p_{CDKFzr}	3.0	$r_{CDKBaFzr}$	0.18	2/2
p_{CDKERb}	2.0	$a_{CDKBaStgi}$	0.18	1/2	p_{CDKERb}	2.0	$a_{CDKBaStgi}$	0.18	2/2
$p_{CDKEDapE2F2}$	4.4	$a_{CDKBaWee}$	0.18	2/2	$p_{CDKEDapE2F2}$	4.4	$a_{CDKBaWee}$	0.18	2/2
$p_{CDKAStgi}$	1.0	$a_{CDKBaE2F}$	0.24	0/2	$p_{CDKAStgi}$	1.0	$a_{CDKBaE2F}$	0.2	2/2
$p_{CDKBaStgi}$	1.5	$a_{StgaCDKBi}$	0.155	0/2	$p_{CDKBaStgi}$	1.5	$a_{StgaCDKBi}$	0.155	1/2
$p_{dephoRbp}$	1.5	$a_{WeeCDKBa}$	0.5	1/1	$p_{dephoRbp}$	1.5	$a_{WeeCDKBa}$	0.5	1/1
$K_{dephoRbp}$	0.1	a_{PlxFzy}	0.3	1/2	$K_{dephoRbp}$	0.1	a_{PlxFzy}	0.3	1/2
$u_{Skp2Dap}$	2.0	$r_{DapE2F2E2F1}$	0.2	0/0	$u_{Skp2Dap}$	2.0	$r_{DapE2F2E2F1}$	0.2	1/0
$u_{Skp2CycE}$	1.5	$r_{Dap2E2F2CycA}$	0.2	1/1	$u_{Skp2CycE}$	1.5	$r_{Dap2E2F2CycA}$	0.25	1/1
$u_{Skp2CDKE}$	4.0	$r_{Dap2E2F2CycB}$	0.2	1/1	$u_{Skp2CDKE}$	4.0	$r_{Dap2E2F2CycB}$	0.25	1/1

Supplementary Table 4 Initial conditions (IC)

Variable	Original IC	IC for identifying a fixed point
CycE	random()/2607374180	0.2179
CDKE	0.0	0.1697
Dap	0.0	0.39
CDKEDap	0.0	0.0993
Skp2	0.0	0.866
E2F1	random()/2607374180	0.2284
Rb	0.0	0.4952
Rbp	0.0	0.0095
RbE2F1	0.0	0.5163
E2F2	0.0	0.4
DapE2F2	0.0	0.1984
CycA	0.0	0.0533
CDKA	0.0	0.1397
Rux	0.0	0.9692
CDKARux	0.0	0.2023
CycB	0.0	0.1713
CDKBi	0.0	0.1857
CDKBa	0.0	0.1879
Wee	0.0	0.4852
Stgi	0.0	0.1176
Stga	0.0	0.1819
Plx	0.0	0.2894
APC	1.0	0.7546
APCFzy	0.0	0.0947
APCFzr	0.0	0.1507

3 Robustness and bifurcation analysis

Bifurcation analysis has been widely used to analyze the qualitative dynamics of the cell cycle control system (Tyson et al 2002). Previous studies have revealed hysteresis and bistability of the system caused by cell mass, CycA, CycB, and cycle number (Csikasz-Nagy et al 2006; Gerard and Goldbeter 2009; Pomerening et al 2003; Calzone et al 2007). In this work we focused on the influence of half-maximal activating and inhibiting coefficients (in Hill functions, which control the timing of events) in the cell cycle control system, especially E2F1-related events, because E2F1 is the core of regulatory feedbacks, and E2F2-related events, because this negative E2F protein is firstly quantitatively examined in this model.

We used the program oscill8 and the model's default parameters to perform bifurcation analysis. First, we found that many half-maximal activating and inhibiting coefficients do not cause the model to generate hysteresis and bistability indicated by the CycE level, that fixed points occur when these coefficients are near their default values, and, especially, that Fzr-related coefficients show no influence on CycE (Supplementary Figure 6). Second, the responses of CycE to changes of $a_{CDKEE2F1}$ and $a_{CDKBaE2F1}$ that control the degradation of E2F1 by CDKE and CDKB, to changes of $a_{E2F1E2F1}$ that controls the activation of E2F1 by E2F1, and to changes of d_{E2F1} that controls the decay of E2F1, show hysteresis (Supplementary Figure 8). Third, DapE2F2 negatively regulates the production of E2F1, CycA, and CycB. Only changes of $r_{DapE2F2E2F1}$ make the model generate complex CycE responses (Supplementary Figure 7). We also examined the responses of E2F1, CycA, and CycB to changes of $r_{DapE2F2E2F1}$, $r_{DapE2F2CycA}$, and $r_{DapE2F2CycB}$, and found that $r_{DapE2F2E2F1}$ makes not only CycE but also CycA generate hysteresis-like responses. Equation (12) indicates that the production of CycA is sensitive to both E2F1 and E2F2. Together, these bifurcation analyses suggest that E2F1 and E2F2 decisively influence the dynamic properties of the cell cycle control system.

Next, we analyzed the robustness of the model against changed timing of signaling events. By running the model with the second and third set of parameters we repeated the analysis shown in Table 3 and generated Supplementary Table 3, in which ↗ and ↘ indicate 66.6% increase and decrease of the control parameter, 0 indicates that oscillating protein concentrations were not generated (cell cycle fails), 1 indicates that oscillating protein concentrations were generated but some events were absent or present persistently, and 2 indicates that oscillating protein concentrations were generated and all signaling events occurred periodically. Thus, the result of 0/0 indicates that the model is highly sensitive to the changed timing of the event, and the result of 2/2 indicates that the model is robust against the changed timing of the event.

We classified all events into 9 groups (Supplementary Table 5). For example, the Fzy group includes *APCFzy_Ubi_CycA*, *APCFzy_Ubi_CDKA*, *APCFzy_Ubi_CycB*, *APCFzy_Ubi_CDKBi*, and *APCFzy_Ubi_CDKBa*. We also quantified the model's responses to changed timing of events by ranking 0/0=1, 0/1=2, 0/2=3, 1/1=4, 1/2=5, 2/2=6. Data generated by the three parameter sets show that protein proteolysis of cyclins and Cdk complexes conducted by Fzy and Fzr are more robust than other events (especially compared with the DapE2F2-related events and Skp2-related events) (Supplementary Table 5). To confirm this, we performed

the global F test. The result of 10.87>2.03 indicates that means are not equal (Supplementary Table 6), or the system's responses to changes of different events show different robustness. We then used methods of multiple comparisons to find where the exact differences lie. Tamhane's T2 multiple comparison test indicates that the means of the Fzy and Fzr groups are significantly larger than the means of the CDKE, Skp2, and E2F2 groups (significance level=0.05). Dunnett T3 test and Games-Howell test generate the same results, in addition that the means of the CDKB group is also large. Although Levene's test indicates that not all populations have a common variance, the Student-Newman-Keuls test and Duncan's multiple range test equally indicate that Fzy, Fzr, and CDKBa are in the same group whose means are significantly larger than others. These statistics therefore support that the proteolysis of Cyclin and Cdk complexes by Fzr and Fzy are more robust compared with other events.

Supplementary Table 5 Robustness against changes of events

**Fzy**	**Fzr**	**CDKB**	**CDKA**	**CDKE**	**E2F1**	**DapE2F2**	**Skp2**	**Wee/Stg**	**Summary**
6	6	2	2	2	2	2	4	5	
6	5	6	4	4	4	4	2	5	
6	5	6	2	4	2	4	2		
6	6	6	2	2	4				
6	6	5		1					
	2								
6	3	3	6	5	2	1	2	4	
6	6	5	3	5	5	4	1	3	
6	6	6	5	4	5	4	2		
6	6	3	6	3	4				
6	6	6		2					
	6								
6	4	6	6	5	3	2	4	4	
6	6	6	5	5	5	4	1	5	
6	6	6	6	2	5	4	4		
6	6	6	6	3	5				
6	6	6		4					
	6								
T=90	T=97	T=78	T=53	T=51	T=46	T=29	T=22	T=26	T=492
N=15	N=18	N=15	N=12	N=15	N=12	N=9	N=9	N=6	N=111
X=6	X=5.389	X=5.2	X=4.4167	X=3.4	X=3.833	X=3.222	X=2.444	X=4.333	X=4.432
s^2=0	s^2=1.428	s^2=1.886	s^2=2.992	s^2=1.829	s^2=1.606	s^2=1.444	s^2=1.528	s^2=0.667	

Supplementary Table 6 The analysis of variance table (k=9, N=111)

Source of variation	Sum of squares	df	MS (MS_G and MS_E)	F	c.v (a=0.05)
Between Groups	$SS_{between}$ = 131.1	8	$MS_{between}$/8=16.41	$MS_{between}/MS_{within}$ = 10.87	2.03

Within Groups	SS_{within} = 153.975$_r$	102	MS_{within}/(102)=1.51		
Total	SS_{total} = 285.24	110			

4 Supplementary Figures

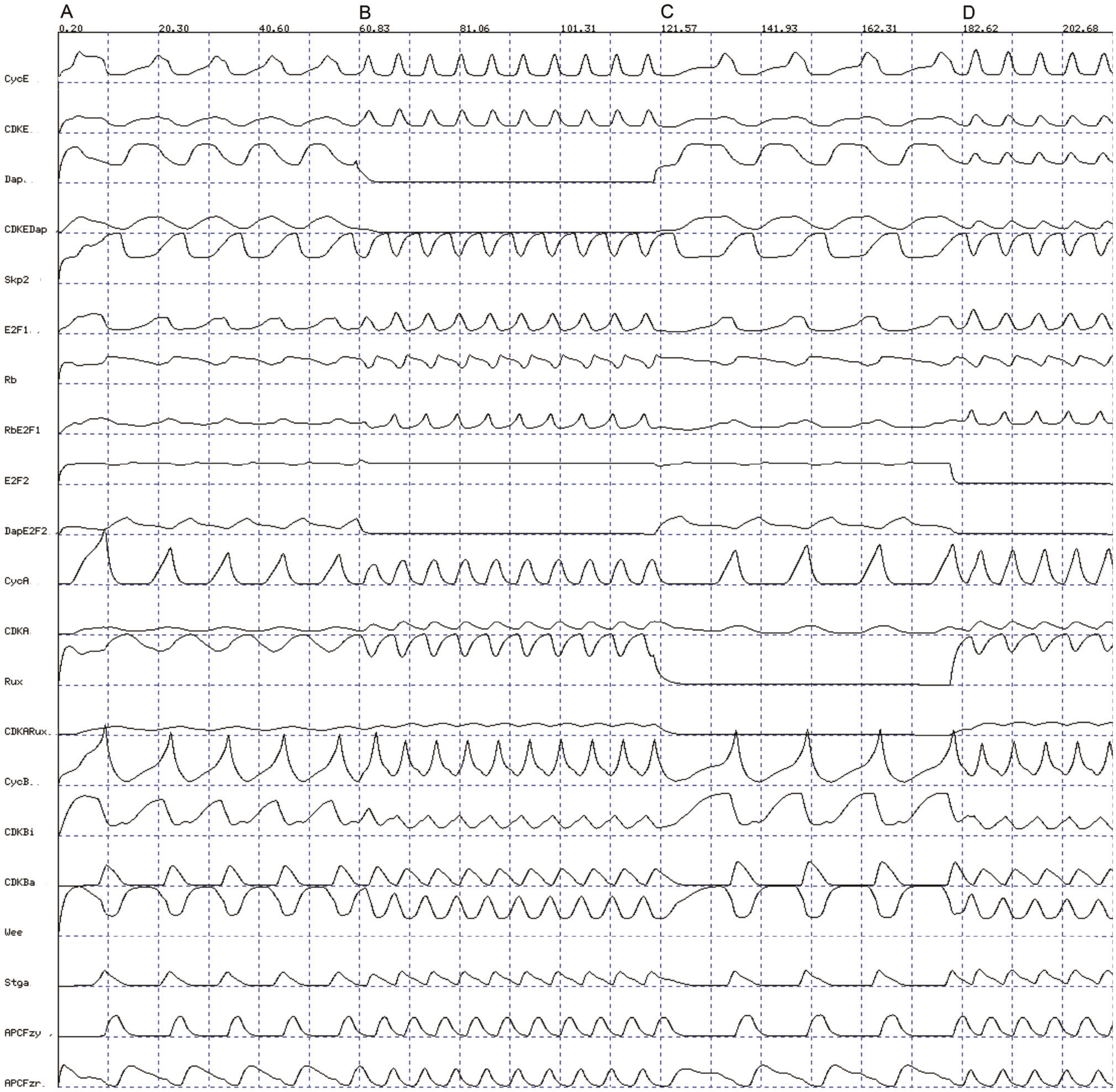

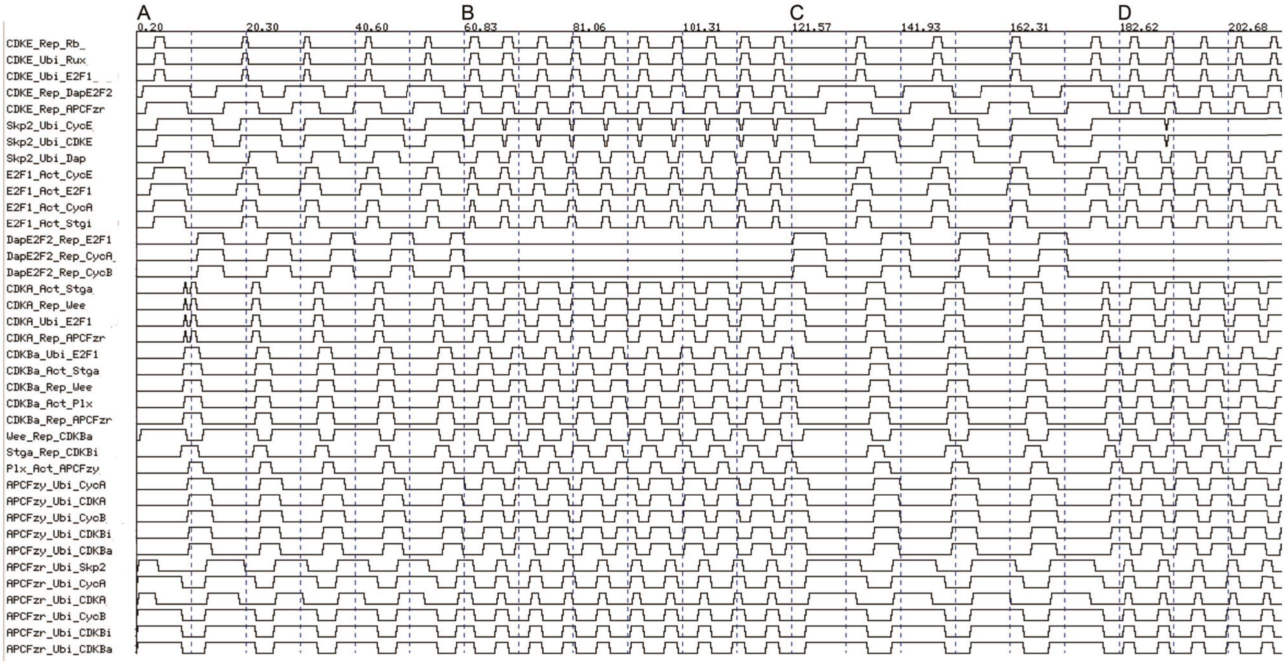

Supplementary Figure 1 The model can produce cell cycles under default parameters (A), and with the absence of Dap (B), Rux (C), or E2F2 (D). The top and bottom panels show protein concentrations and signaling events.

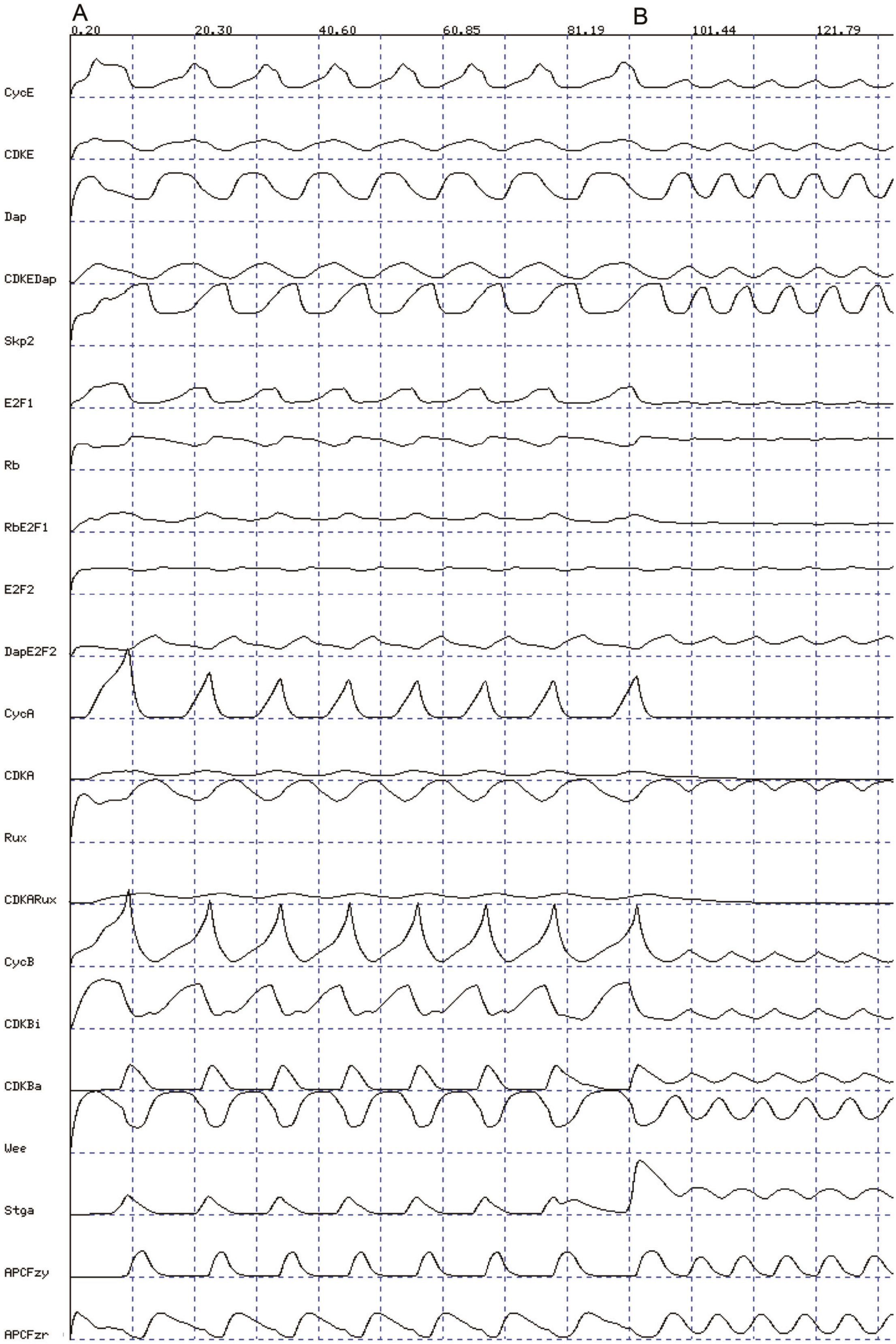

Supplementary Figure 2 High level of Stg arrests cell cycles. (A) Default parameters. (B) dStga=1.0→0.3.

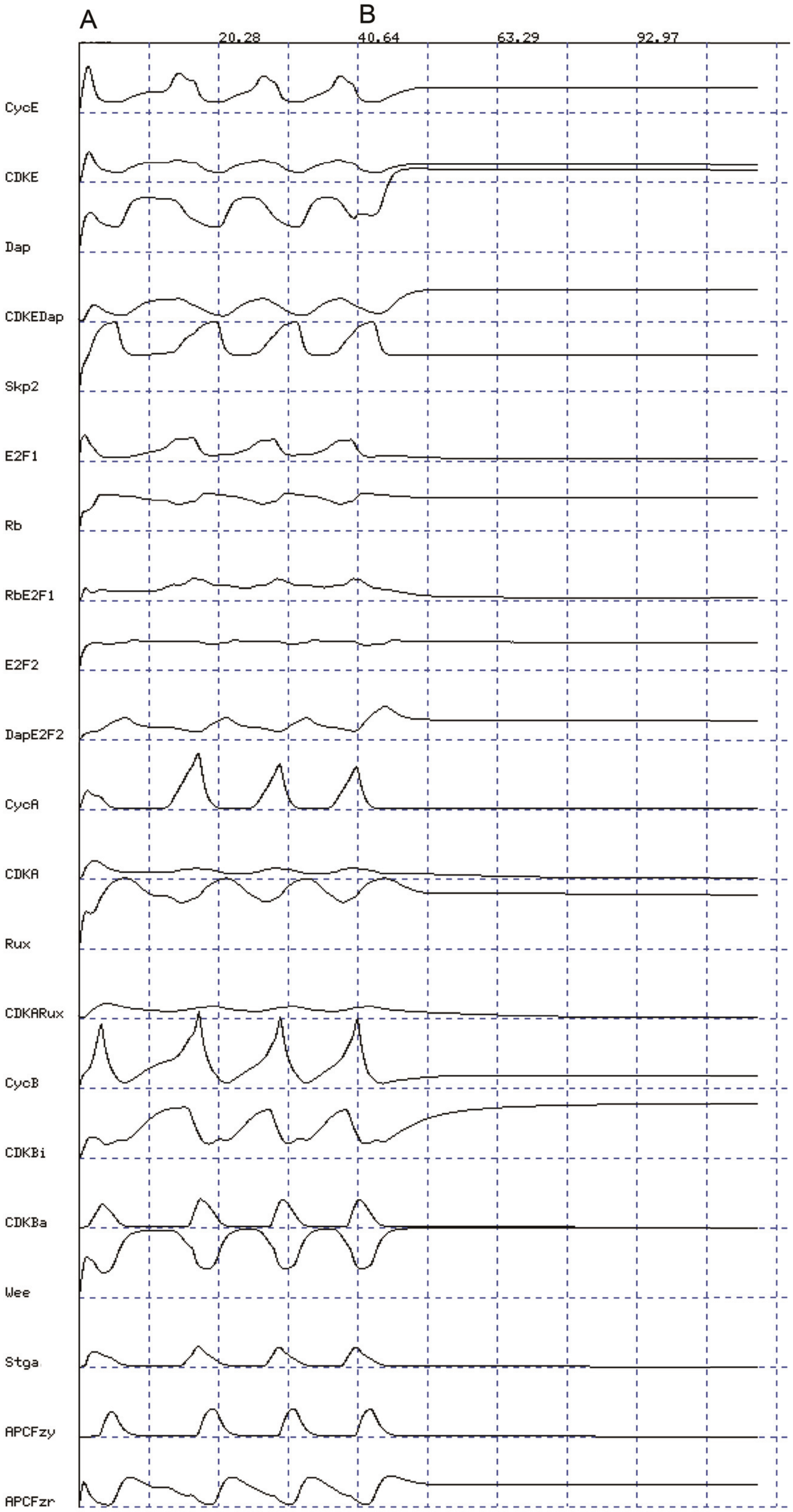

Supplementary Figure 3 High level of Dap alone causes cell cycle arrest. (A) Default parameters. (B) sDap=1.0→1.5.

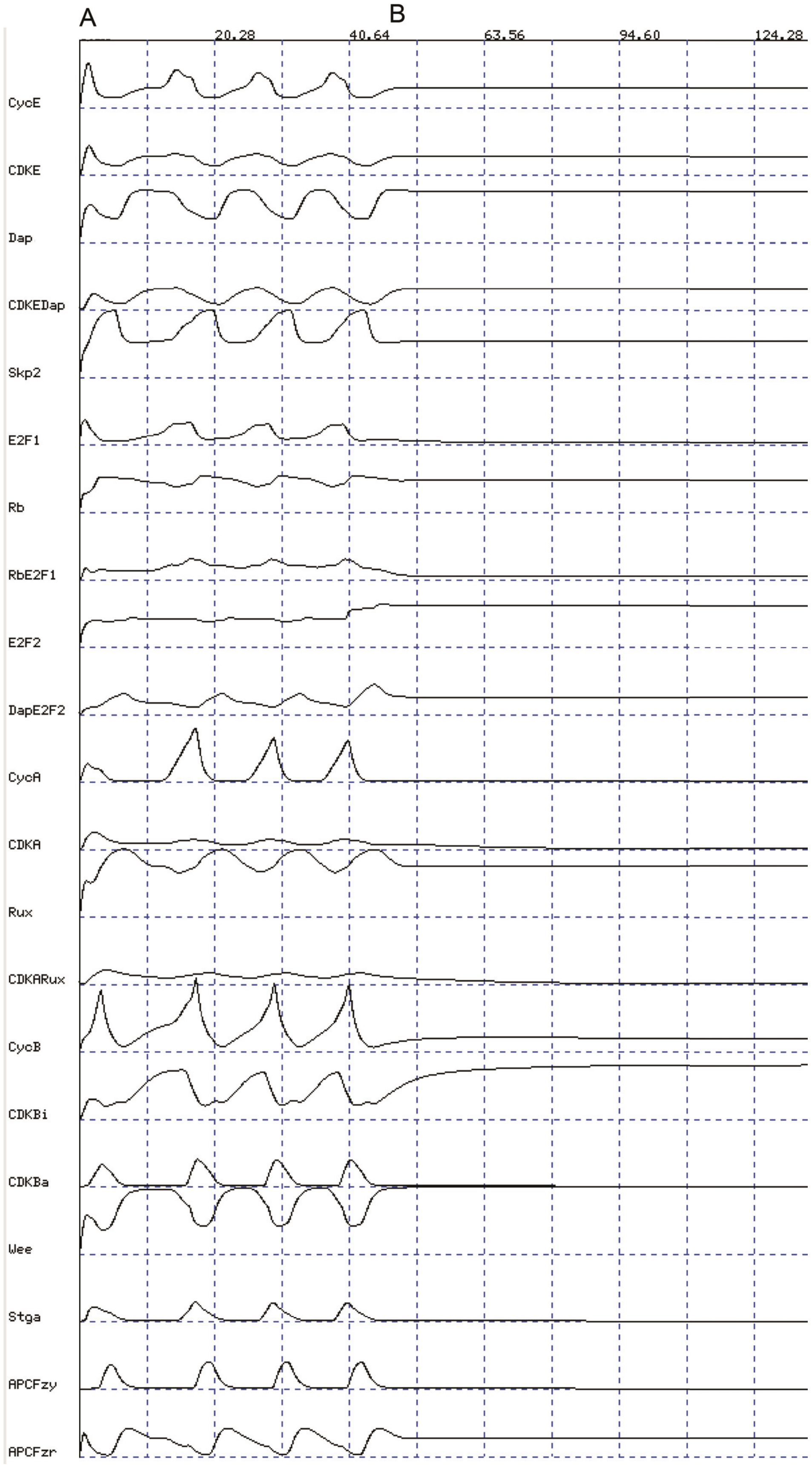

Supplementary Figure 4 High level of E2F2 alone causes cell cycle arrest. (A) Default parameters. (B) sE2F2=1.0→1.5.

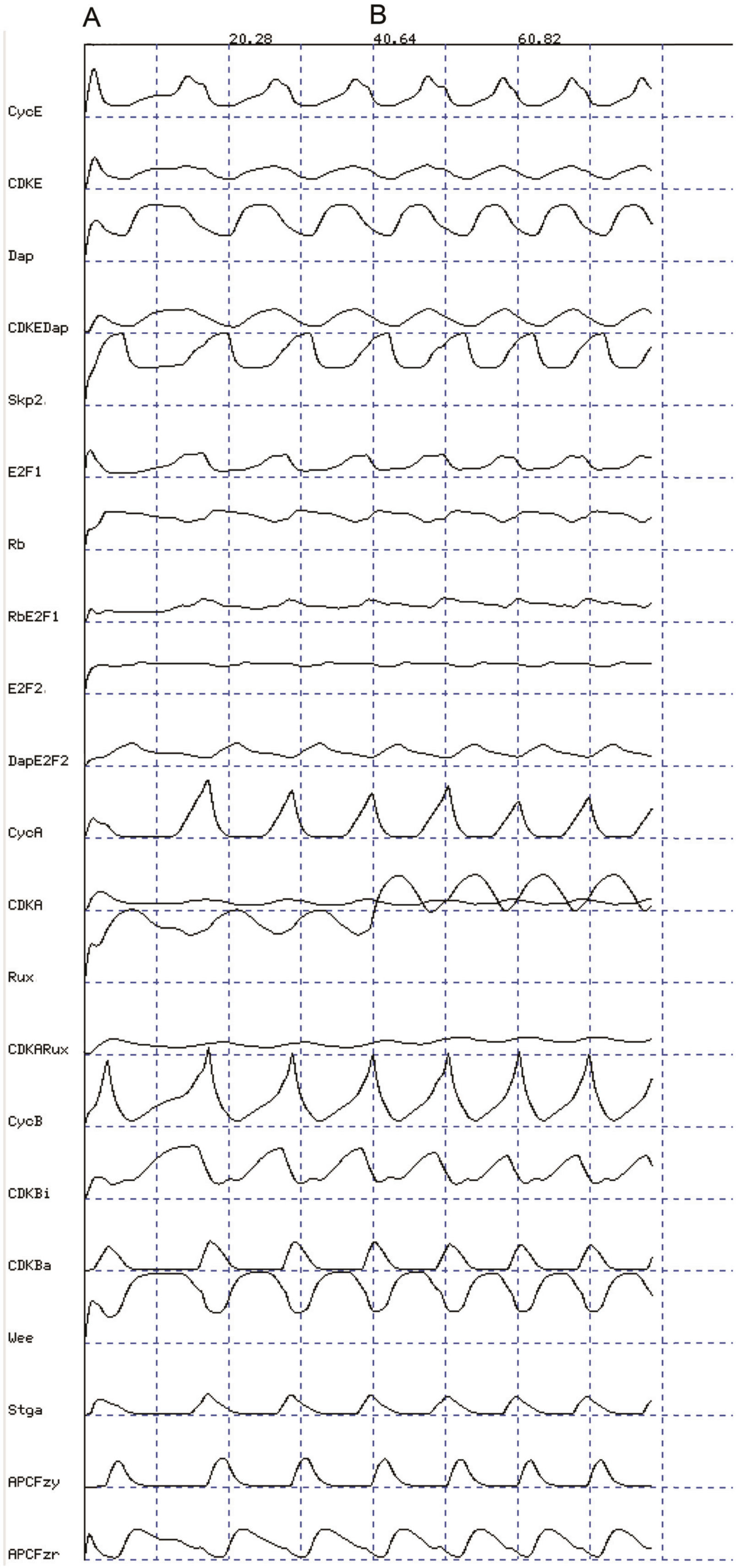

Supplementary Figure 5 The system is more tolerable for the increase of Rux. (A) Default parameters. (B) sRux=1.0→1.5.

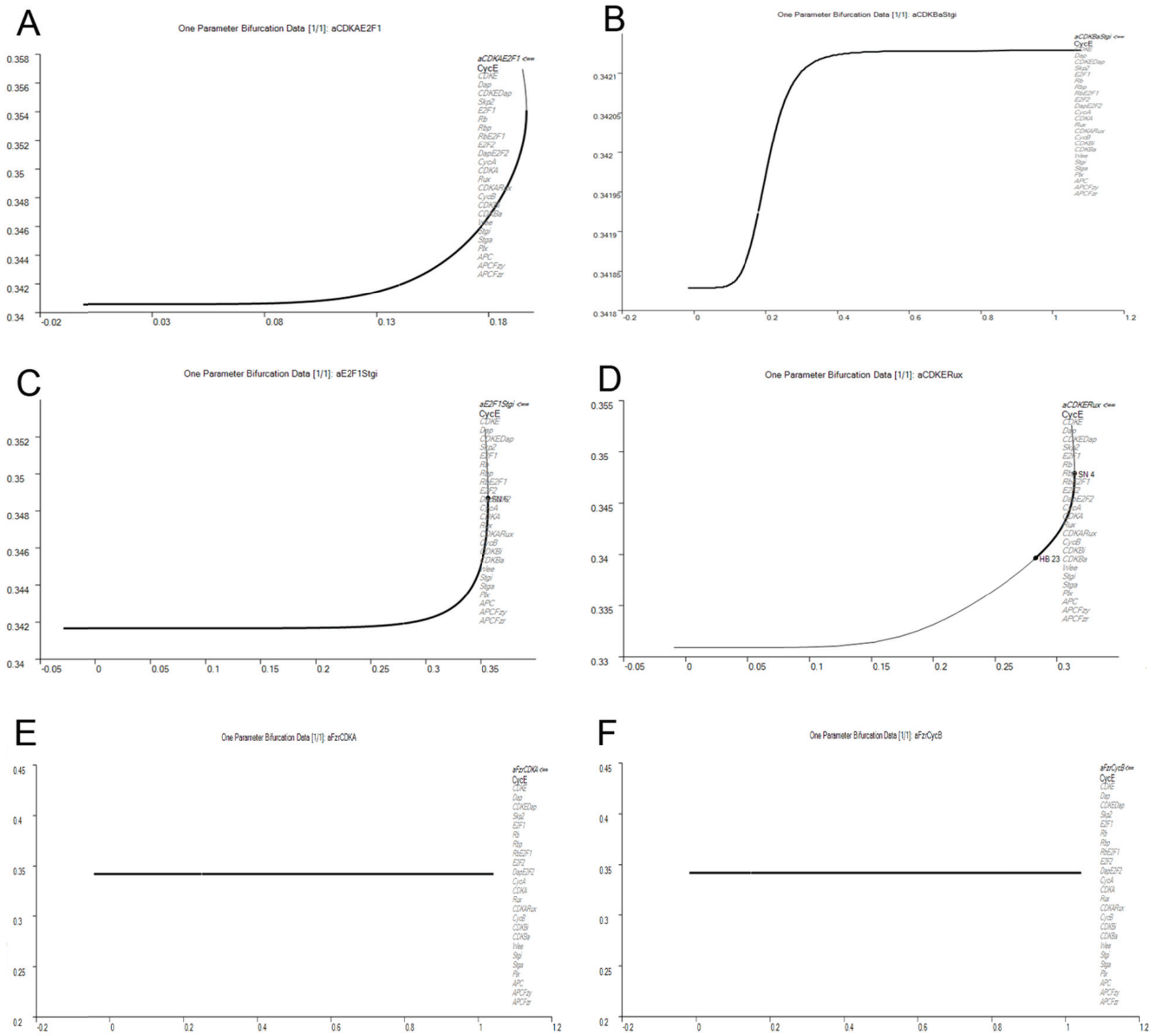

Supplementary Figure 6 One-parameter bifurcation diagrams for the parameter $a_{CDKAE2F1}$ (A), $a_{CDKBaStigi}$ (B), $a_{E2F1Stgi}$ (C), $a_{CDKERux}$ (D), $a_{FzrCDKA}$ (E), and $a_{FzrCycB}$ (F). CycE is the variable. In these and other bifurcation diagrams, thick and thin lines indicate stable and unstable regions of fixed points.

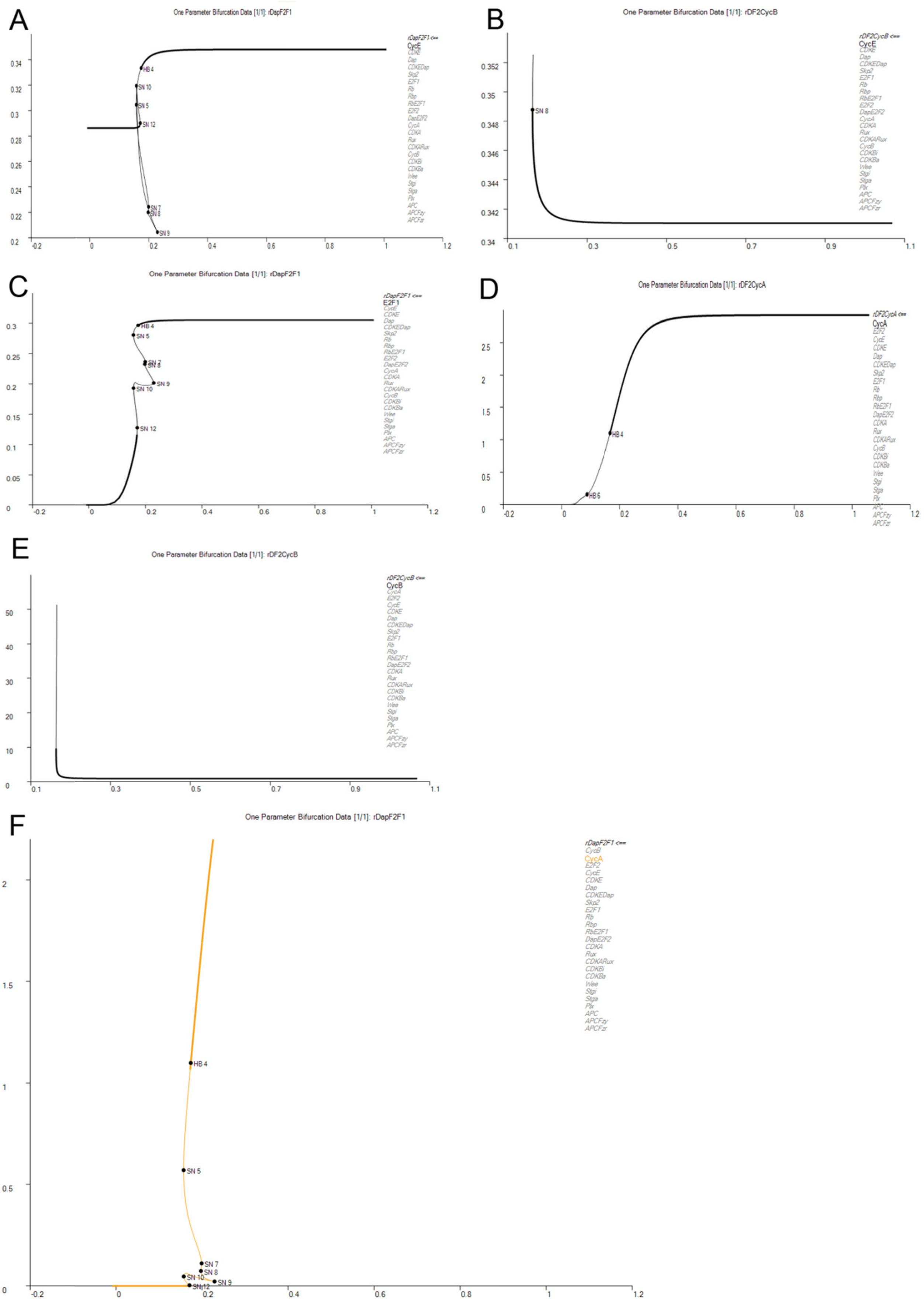
A
One Parameter Bifurcation Data [1/1]: rDapF2F1
rDapF2F1 <==
CycE
HB 4
SN 10
SN 5
SN 12
SN 7
SN 8
SN 9
B
One Parameter Bifurcation Data [1/1]: rDF2CycB
rDF2CycB <==
CycE
SN 8
C
One Parameter Bifurcation Data [1/1]: rDapF2F1
rDapF2F1 <==
E2F1
HB 4
SN 5
SN 7
SN 8
SN 9
SN 10
SN 12
D
One Parameter Bifurcation Data [1/1]: rDF2CycA
rDF2CycA <==
CycA
HB 4
HB 6
E
One Parameter Bifurcation Data [1/1]: rDF2CycB
rDF2CycB <==
CycB
F
One Parameter Bifurcation Data [1/1]: rDapF2F1
rDapF2F1 <==
CycA
HB 4
SN 5
SN 7
SN 8
SN 10
SN 9
SN 12

Supplementary Figure 7 One-parameter bifurcation diagrams for the parameter $r_{DapE2F2E2F1}$ and variable CycE (A), parameter $r_{DapE2F2CycB}$ and variable CycE (B), parameter $r_{DapE2F2E2F1}$ and variable E2F1 (C), parameter $r_{DapE2F2CycA}$ and variable CycA (D), parameter $r_{DapE2F2CycB}$ and variable CycB (E), and parameter $r_{DapE2F2E2F1}$ and variable CycA (F).

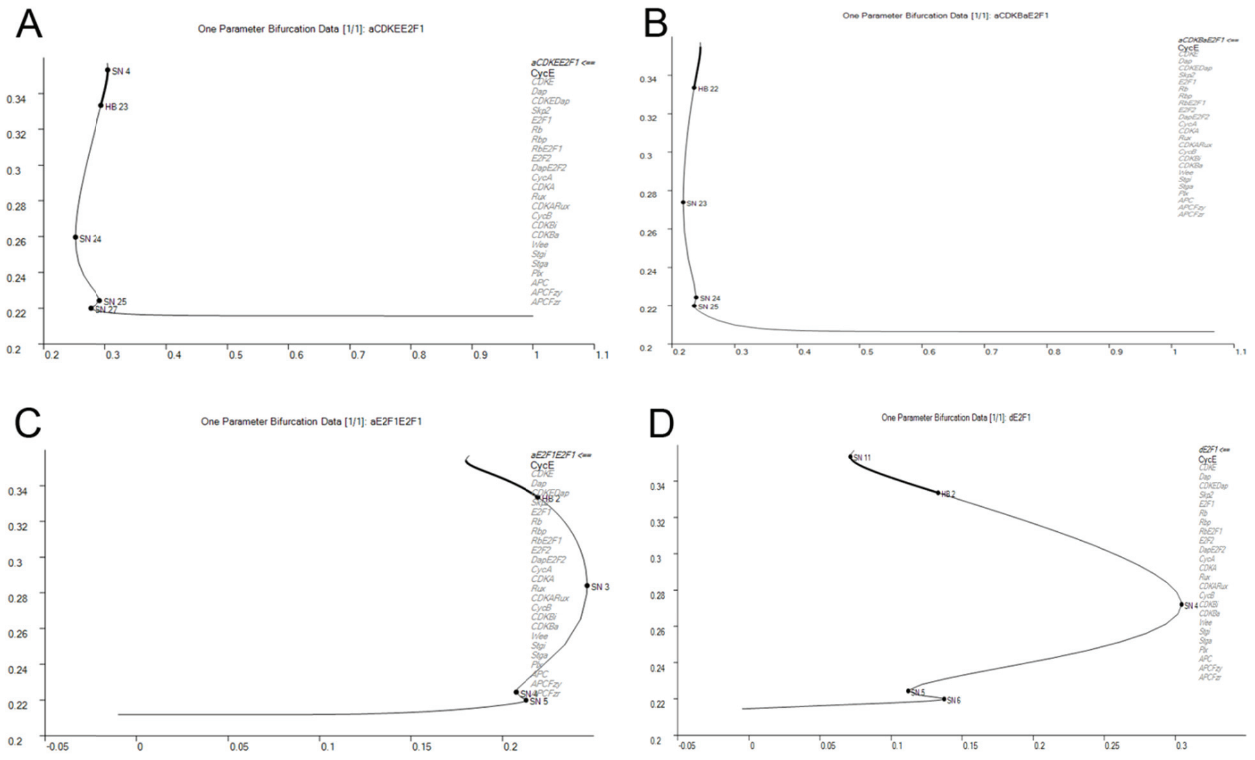

Supplementary Figure 8 One-parameter bifurcation diagrams for the parameter $a_{CDKEE2F1}$ (A), $a_{CDKBaE2F1}$ (B), $a_{E2F1E2F1}$ (C), d_{E2F1} (D). CycE is the variable.

Supplementary References

Amati B, Vlach J. Kip1 meets SKP2: new links in cell-cycle control. *Nat Cell Biol 1999,*1:E91–E93

Avedisov SN, Krasnoselskata I, Mortin M, Thomas BJ. Roughex mediates G1 arrest through a physical association with cyclin A. *Mol Cel Biol* 2000, 20:8220-8229

Baker NE. Patterning signals and proliferation in Drosophila imaginal discs. *Current Opinion in Genetics & Development* 2007, 17:287–293

Barik D, Baumann WT, Paul MR, Novak B, Tyson JJ. A model of yeast cell-cycle regulation based on multisite phosphorylation. *Mol Syst Biol* 2010 6:405

Bashir T, Dorrello NV, Amador V, Guardavaccaro D, Pagano M. Control of the SCFSkp2–Cks1 ubiquitin ligase by the APC/CCdh1 ubiquitin ligase. *Nature* 2004,428:190-193.

Calzone L, Thieffry D, Tyson JJ, Novak B. Dynamical modeling of syncytial mitotic cycles in Drosophila embryos. Molecular Systems Biology 2007, 3:131.

Chen KC, Calzone L, Csikasz-Nagy A, Cross FR, Novak B, Tyson JJ. Integrative analysis of cell cycle control in budding yeast. *Molecular Biology of the Cell* 2004,15:3841-3862

Csikasz-Nagy A, Battogtokh D, Chen KC, Novak B, Tyson JJ. Analysis of a generic model of eukaryotic cell-cycle regulation. *Biophysical J* 2006, 90:4361–4379

Escudero LM, Freeman M. Mechanism of G1 arrest in the *Drosophila* eye imaginal disc. *BMC Dev Biol* 2007, 7:13

de Nooij JC, Letendre MA, Hariharan IK. A cyclin-dependent kinase inhibitor, Dacapo, is necessary for timely exit from the cell cycle during Drosophila embryogenesis. *Cell* 1996, 87:1237–1247.

Dui W, Wei B, He F, Lu W, Li C, Liang X, Ma J, Jiao R. The *Drosophila* F-box protein dSkp2 regulates cell proliferation by targeting Dacapo for degradation. *Molecular Biology of the Cell* 2013, 24:1676-1687

Felix MA, Labbe JC, Doree M, Hunt T, Karsenti E. Triggering of cyclin degradation in interphase extracts of amphibian eggs by cdc2 kinase. *Nature* 1990, 346:379-382

Foley E, O'Farrell PH, Sprenger F. Rux is a cyclin-dependent kinase inhibitor (CKI) specific for mitotic cyclin–Cdk complexes. *Curr Biol* 1999, 9:1392–1402

Fung TK, Ma HT, Poon RYC. Specialized roles of the two mitotic cyclins in somatic cells:Cyclin A as an activator of M phase–promoting factor. *Molecular Biology of the Cell* 2007, 18:1861–

1873

Gerard C, Goldbeter A. Temporal self-organization of the cyclin/Cdk network driving the mammalian cell cycle. *Proc Natl Sci Acad USA* 2009, 106:21643–21648

Guardavaccaro D , Pagano M. Stabilizers and destabilizers controlling cell cycle oscillators. *Mol Cell* 2006, 22:1-4

Haberichter T, Madge B, Christopher RA, Yoshioka N, Dhiman A, Miller R, Gendelman R, Aksenov SV, Khalil IG, Dowdy SF. A systems biology dynamical model of mammalian G1 cell cycle progression. *Mol Syst Biol* 2007, 3:84

Havens CG, Walter JC. Docking of a specialized PIP Box onto chromatin-bound PCNA creates a degron for the ubiquitin ligase CRL4Cdt2. *Mol Cell* 2009, 35:93–104

Havens CG, Walter JC. Mechanism of CRL4(Cdt2), a PCNA-dependent E3 ubiquitin ligase. *Genes Dev*. 2011, 25:1568-1582

Hochegger H, Takeda S, Hunt T. Cyclin-dependent kinases and cell-cycle transitions: does one fit all? *Nat Rev Mol Cell Biol* 2008,9:910-916

Kim SY, Ferrell, Jr. JE. Substrate competition as a source of ultrasensitivity in the inactivation of Wee1. *Cell* 2007, 128:1133–1145.

Kitagawa M, Higashi H, Suzuki-Takahashi I, Segawa K, Hanks SK, Taya Y, Nishimura S, Okuyama A. Phosphorylation of E2F-1 by cyclin A-cdk2. *Oncogene* 1995,10:229-236

Kolupaeva V, Janssens V. PP1 and PP2A phosphatases--cooperating partners in modulating retinoblastoma protein activation. *FEBS J* 2013 280:627-643

Lehman DA, Patterson B, Johnston LA, Balzer T, Britton JS, Saint R, Edgar BA. Cis-regulatory elements of the mitotic regulator, string/Cdc25. *Development* 1999, 126:1793-1803

Macaluso M, Montanari M, Giordano A. Rb family proteins as modulators of gene expression and new aspects regarding the interaction with chromatin remodeling enzymes. *Oncogene* 2006, 25:5263–5267

Malumbres M, Barbacid M. Mammalian cyclin-dependent kinases. *TRENDS in Biochemical Sciences* 2006, 30:630-641

Moberg KH, Bell DW, Wahrer DCR, Haber DA, Hariharan IK. Archipelago regulates Cyclin E levels in Drosophila and ismutated in human cancer cell lines. *Natuer* 2001,413:311-316

Morgan DO. The cell cycle: Principle of control. Oxford University Press 2007

Narbonne-Reveau K, Senger S1, Pal M, Herr A, Richardson HE, Asano M, Deak P, Lilly MA.†APC/CFzr/Cdh1 promotes cell cycle progression during the *Drosophila* endocycle. *Development* 2008, 135:1451-1461

Novak B, Tyson JJ. A model for restriction point control of the mammalian cell cycle. *J Theor Biol* 2004, 230:563–579

Pippa R, Espinosa L, Gundem G, Garca-Escudero R, Dominguez A, Orlando S, Gallastegui E, Saiz C, Besson A, Pujol MJ, Lopez-Bigas N, Paramio JM, Bigas A, Bachs O. p27Kip1 represses transcription by direct interaction with p130/E2F4 at the promoters of target genes. *Oncogene* 2012, 31:4207–4220

Plesca D, Crosby ME, Gupta D, Almasan A. E2F4 function in G2: Maintaining G2-arrest to prevent mitotic entry with damaged DNA. *Cell Cycle*. 2007, 6:1147–1152

Pomerening, J. R., Sontag, E. D. & Ferrell, J. E., Jr. Building a cell cycle oscillator: hysteresis and bistability in the activation of Cdc2. *Nat Cell Biol* **5**, 346-351, doi:10.1038/ncb954 (2003).

Raff JW, Jeffers K, Huang J-Y. The roles of Fzy/Cdc20 and Fzr/Cdh1 in regulating the destruction of cyclin B in space and time. *The Journal of Cell Biology* 2002, 157:1139–1149

Rayman JB, Takahashi Y, Indjeian VB, Dannenberg J-H, Catchpole S, Watson RJ, te Riele H, Dynlacht BD. E2F mediates cell cycle-dependent transcriptional repression in vivo by recruitment of an HDAC1/mSin3B corepressor complex. *Genes Dev* 2002, 16:933–947

Reis T, Edgar BA. Negative regulation of dE2F1 by cyclin-dependent kinases controls cell cycle timing. *Cell* 2004, 117:253-264

Rossi M, Duan S, Jeong YT, Horn M, Saraf A, Florens L, Washburn MP, Antebi A, Pagano M. Regulation of the CRL4(Cdt2) ubiquitin ligase and cell-cycle exit by the SCF (Fbxo11) ubiquitinligase. *Mol Cell* 2013, 49:1159-1166

Shibutani ST, de la Cruz AF, Tran V, Turbyfill WJ 3rd, Reis T, Edgar BA, Duronio RJ. Intrinsic_negative_cell cycle regulation_provided_by PIP box- and Cul4Cdt2-mediated destruction of E2f1 during S phase. *Dev Cell* 2008,15:890-900

Thomas BJ, Zavitz KH, Dong X, Lane ME, Weigmann K, Finley Jr. RL, Brent R, Lehner CF, Zipursky SL. *roughex* down-regulates G2 cyclins in G1. *Genes Dev* 1997, 11:1289-1298

Trunnell NB, Poon AC, Kim SY, Ferrell, Jr. JE. Ultrasensitivity in the regulation of Cdc25C by Cdk1. *Mol Cell* 2011, 41:263–274

Tsai TY, Choi YS, Ma W, Pomerening JR, Tang C, Ferrell Jr JE. Robust, tunable biological oscillations from interlinked positive and negative feedback loops. *Science* 2008,321:126-129

Tyson JJ, Novak B. Temporal organization of the cell cycle. *Curr Biol* 2008, 18:R759-R768.

Tyson JJ, Csikasz-Nagy A, Novak B. The dynamics of cell cycle regulation. BioEssays 2002, 24:1095-1109.

Watanabe N, Arai H, Iwasaki J, Shiina M, Ogata K, Hunter T, Osada H. Cyclin-dependent kinase (CDK) phosphorylation destabilizes somatic Wee1 via multiple pathways. *Proc Natl Acad Sci USA* 2005, 102:11663–11668

Wei W, Ayad NG, Wan Y, Zhang G-J, Kirschner MW, Kaelin Jr WG. Degradation of the SCF component Skp2 in cell-cycle phase G1 by the anaphase-promoting complex. *Nature* 2004,428:194-198

Wolthuis R, Clay-Farrace L, van Zon W, Yekezare M, Koop L, Ogink J, Medema R, Pines J. Cdc20 and Cks direct the spindle checkpoint-independent destruction of cyclin A. *Mol Cell* 2008, 30:290–302

Xu M, Sheppard K-A, Peng C-Y, Yee AS, Piwnica-Worms H. Cyclin A/CDK2 binds directly to E2F-1 and inhibits the DNA-binding activity of E2F-1/DP-1 by phosphorylation. *Mol Cell Biol* 1994, 12:8420-8431

Yang Q, Ferrell Jr JE. The Cdk1_APC/C cell cycle oscillator circuit functions as a time-delayed, ultrasensitive switch. *Nat Cell Biol* 2013, 15:519-525

Yang L, Han Z, MacLellan WB, Weiss JN, Qu Z. Linking cell division to cell growth in a spatiotemporal model of the cell cycle. *J Theor Biol* 2006, 241:120–133.

Yao G, Lee TJ, Mori S, Nevins JR, You L. A bistable Rb–E2F switch underlies the restriction point. *Nat Cell Biol* 2008,10:476-482

Yao G, Tan C, West M, Nevins JR, You L. Origin of bistability underlying mammalian cell cycle entry. *Mol Syst Biol* 2011,7:485

Zielke N, Querings S, Rottig C, Lehner C, Sprenger F. The anaphase-promoting complex/cyclosome (APC/C) is required for rereplication control in endoreplication cycles. *Genes Dev* 2008, 22:1690–1703

Zielke N, Kim KJ, Tran V, Shibutani ST, Bravo MJ, Nagarajan S, van Straaten M, Woods B, von Dassow G, Rottig C, Lehner CF, Grewal SS, Duronio RJ, Edgar BA. Control_of_Drosophila endocycles_by_E2F_and CRL4(CDT2). *Nature* 2011, 480:123-127
